# Soybean (*Glycine max*) expansin gene superfamily origins: segmental and tandem duplication events followed by divergent selection among subfamilies

**DOI:** 10.1186/1471-2229-14-93

**Published:** 2014-04-11

**Authors:** Yan Zhu, Ningning Wu, Wanlu Song, Guangjun Yin, Yajuan Qin, Yueming Yan, Yingkao Hu

**Affiliations:** 1 College of Life Sciences, Capital Normal University, Beijing 100048, China

## Abstract

**Background:**

Expansins are plant cell wall loosening proteins that are involved in cell enlargement and a variety of other developmental processes. The expansin superfamily contains four subfamilies; namely, α-expansin (EXPA), β-expansin (EXPB), expansin-like A (EXLA), and expansin-like B (EXLB). Although the genome sequencing of soybeans is complete, our knowledge about the pattern of expansion and evolutionary history of soybean expansin genes remains limited.

**Results:**

A total of 75 expansin genes were identified in the soybean genome, and grouped into four subfamilies based on their phylogenetic relationships. Structural analysis revealed that the expansin genes are conserved in each subfamily, but are divergent among subfamilies. Furthermore, in soybean and *Arabidopsis*, the expansin gene family has been mainly expanded through tandem and segmental duplications; however, in rice, segmental duplication appears to be the dominant process that generates this superfamily. The transcriptome atlas revealed notable differential expression in either transcript abundance or expression patterns under normal growth conditions. This finding was consistent with the differential distribution of the *cis*-elements in the promoter region, and indicated wide functional divergence in this superfamily. Moreover, some critical amino acids that contribute to functional divergence and positive selection were detected. Finally, site model and branch-site model analysis of positive selection indicated that the soybean expansin gene superfamily is under strong positive selection, and that divergent selection constraints might have influenced the evolution of the four subfamilies.

**Conclusion:**

This study demonstrated that the soybean expansin gene superfamily has expanded through tandem and segmental duplication. Differential expression indicated wide functional divergence in this superfamily. Furthermore, positive selection analysis revealed that divergent selection constraints might have influenced the evolution of the four subfamilies. In conclusion, the results of this study contribute novel detailed information about the molecular evolution of the expansin gene superfamily in soybean.

## Background

Expansins are encoded by a multi-gene family, and are composed of a superfamily of plant cell wall loosening proteins that induce pH-dependent wall extension and stress relaxation in a characteristic and unique manner [1]. Expansins were first identified in studies investigating the mechanism of plant cell wall enlargement, and were isolated from cucumber hypocotyls [2]. Recently, increasing numbers of expansins have been identified in other plant species, including oat [3], tomato [4], and maize [5]. According to the nomenclature proposed by Kende et al. [6], the expansin superfamily in plants may be divided into four subfamilies based on phylogenetic sequence analysis; these subfamilies are designated as α-expansin (EXPA), β-expansin (EXPB), expansin-like A (EXLA), and expansin-like B (EXLB). α-Expansin and β-expansin proteins are known to exhibit cell wall loosening activity, and are involved in cell expansion and other developmental events; however, expansin-like A and expansin-like B are only known from their gene sequences [7], with no experimental evidence about their activity on the cell wall being published [8].

Functional studies have shown that expansins are involved in many developmental processes, such as fruit softening [9], xylem formation [10], abscission (leaf shedding) [11], seed germination [12], and the penetration of pollen tubes [13,14]. The plant cell wall is composed of cellulose microfibrils, which bind to various glycans, including xyloglucan and xylan. The extension of the cell wall involves the movement and separation of cellulose microfibrils by the process of molecular creeping. α-Expansinis hypothesized to promote such movement, by inducing the local dissociation and slippage of xyloglucans, whereas β-expansin is theorized to work in a similar manner on a different glycan, perhaps xylan [7]. However, no assays have demonstrated that expansins have hydrolytic activity or any other enzymatic activities [15-17].

Expansin proteins are typically 250–275 amino acids long, and contain two domains that are preceded by a signal peptide of 20–30 amino acids in length [7]. Domain I has significant, but distant, homology to glycoside hydrolase family family-45 (GH45) proteins, including a series of conserved cysteines and a His-Phe-Asp (HFD) motif that makes up part of the catalytic site of family-45 endoglucanases [9,18]. Domain II is distantly related to group-2 grass pollen allergens [9]. Domain II is speculated to be a polysaccharide binding domain based on conserved aromatic and polar residues on the surface of the protein [18]. Only the crystal structure of one bacterial expansin [19] and the Zea m 1 in maize [20] have been solved.

The completion of soybean genome sequencing [21] provides us with an opportunity to improve our understanding about the evolution, and other characteristics, of the expansin superfamily in this plant species. In this study, we identified the expansin genes in the soybean genome, and grouped them into four subfamilies. In addition, the expansion patterns of the expansin gene family in *Arabidopsis*, rice, and soybean were examined. The results indicated that expansin genes in soybean are generated through tandem and segmental duplication. Analysis of the transcriptome atlas of soybean expansin genes in different tissues under normal conditions indicated notable differential expression among subfamilies. This finding indicates the presence of broad functional divergence in this superfamily. Critical amino acids that are responsible for functional divergence were detected. In addition, the location of the amino acid sites that are responsible for functional divergence and/or positive selection indicated the conservation of domain I and the C terminus. The results presented in this study are expected to facilitate further research on this gene family, and provide new insights about the evolutionary history of expansins.

## Results

### Genome-wide identification of the expansin gene superfamily in soybean

**Figure 1:**
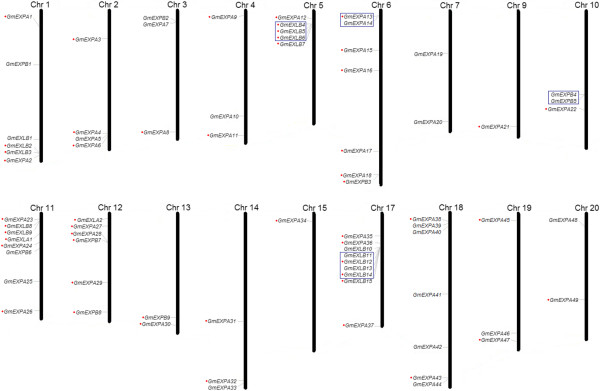
**Chromosomal distribution of soybean (*Glycine max*) expansin genes.** Chromosome size is indicated by its relative length. Chromosomes bearing no expansin genes (Chromosome 8 and 16) are not showed in this figure. Tandemly duplicated genes are represented by boxes with blue outlines. Segmental duplicated genes are indicated by red dots on the leftside. The figure was produced using the Map Inspector program.

Through soybean genome blast and online software identification, a total of 75 soybean expansin genes (Additional file 1) were identified based on expansin nomenclature [6]. All of the 75 members contained the two domains (PF03330 and PF01357) based on Pfam and SMART tests. Proteins that have only one of these domains, or that did not have an integral open reading frame, were excluded. The protein sequences (Additional file 2), coding sequences (CDS) (Additional file 3), genomic sequences (Additional file 4), and 1500 bp of the nucleotide sequences upstream of the translation initiation codon (Additional file 5) were all downloaded from the Phytozome database (http://www.phytozome.com). In addition, the physical positions of the expansin genes were also obtained from the Phytozome database, and were used to map them to their corresponding chromosomes (Figure 1). The results showed that, with the exception of chromosomes 8 and 16, expansin genes could be mapped on all chromosomes from 1 to 20. Chromosome 17 had the highest density of expansin genes, with nine members, whereas chromosome 7, 9, 13, 15, and 20 contained no more than two expansin genes. To clarify which subfamily (EXPA, EXPB, EXLA, or EXLB) these expansin genes belonged to, we employed MEGA v5.0 to construct an unrooted phylogenetic tree using the neighbor-joining (NJ) method, using the entire expansin protein sequences of soybean, *Arabidopsis*, and rice (Additional file 6). Since the expansin genes of *Arabidopsis* and rice have already been classified, we were able to classify the soybean expansin genes according to the clustering exhibited on the phylogenetic tree. The soybean expansin genes were accordingly classified into the four known subfamilies: α-expansin (EXPA), β-expansin (EXPB), expansin-like A (EXLA), and expansin-like B (EXLB). On the basis of the nomenclature rules proposed by Kende et al. [6], we named the 75 expansin genes in soybean using their loci and the subfamily to which they belonged. Basic information on all soybean expansins (including gene name, loci, protein length, signal peptide length, intron number, pI value, and molecular weight) is provided in Additional file 1. The 75 expansins in soybean are 218 ~ 309 amino acids long, with a molecular weight ranging from 23.5 to 33.8kD. All 75 expansins contain signal peptides of 16 to 31 amino acids in length, except for 10 members that lack signal peptides. The pI value ranges from 4.5 to 9.8 in the soybean expansin superfamily, with differences existing between EXLB and other subfamilies. Almost all of the members in the EXPA, EXPB, and EXLA subfamilies have pI values above 7.0, while the pI values of most members in EXLB are below 7.0.

To obtain more information about the size characteristics of the four expansin subfamilies, we compared the expansin genes in five plant species (*Arabidopsis*, rice, soybean, and two other legumes, *Medicago truncatula* and *Phaseolus vulgaris*). Data on the sizes of the four subfamilies in *Arabidopsis* and rice were obtained from a review [7]. In addition, we conducted genome-wide identification of the expansin gene superfamily in *Medicago truncatula* and *Phaseolus vulgaris* (Additional file 7), following the same method used for the identification of the soybean expansin gene superfamily. 36 and 18 expansin genes were identified in the *Phaseolus vulgaris* and *Medicago truncatula*, respectively. We then classified these expansin genes into four subfamilies according to the phylogenetic tree (Additional file 7). The results of the size comparisons of the subfamilies among the five species are shown in Table 1. The distribution of the expansin genes in the four subfamilies was rather uneven. In each of the five species, EXLA had the smallest subfamily size, while EXPA had the largest subfamily size (Table 1). The two legumes, soybean and *Phaseolus vulgaris*, had much larger EXLB subfamilies (with 15 members in soybean and 5 members in *Phaseolus vulgaris*) compared to just one member in both *Arabidopsis* and rice. In contrast, the legume *Medicago truncatula* only had one EXLB member. In addition, the EXPB subfamily was much larger in rice compared to the other four dicot species.

**Table 1:** Sizes of the four expansin subfamilies in different plants

**Species**	**EXPA**	**EXPB**	**EXLA**	**EXLB**
*Arabidopsis	26	6	3	1
*Rice	34	19	4	1
Soybean	49	9	2	15
*Phaseolus vulgaris*	25	6	0	5
*Medicago truncatula*	16	1	0	1

Note: *Datas collected from the review [7].

### Phylogenetic and structural analysis of expansin genes in soybean

**Figure 2:**
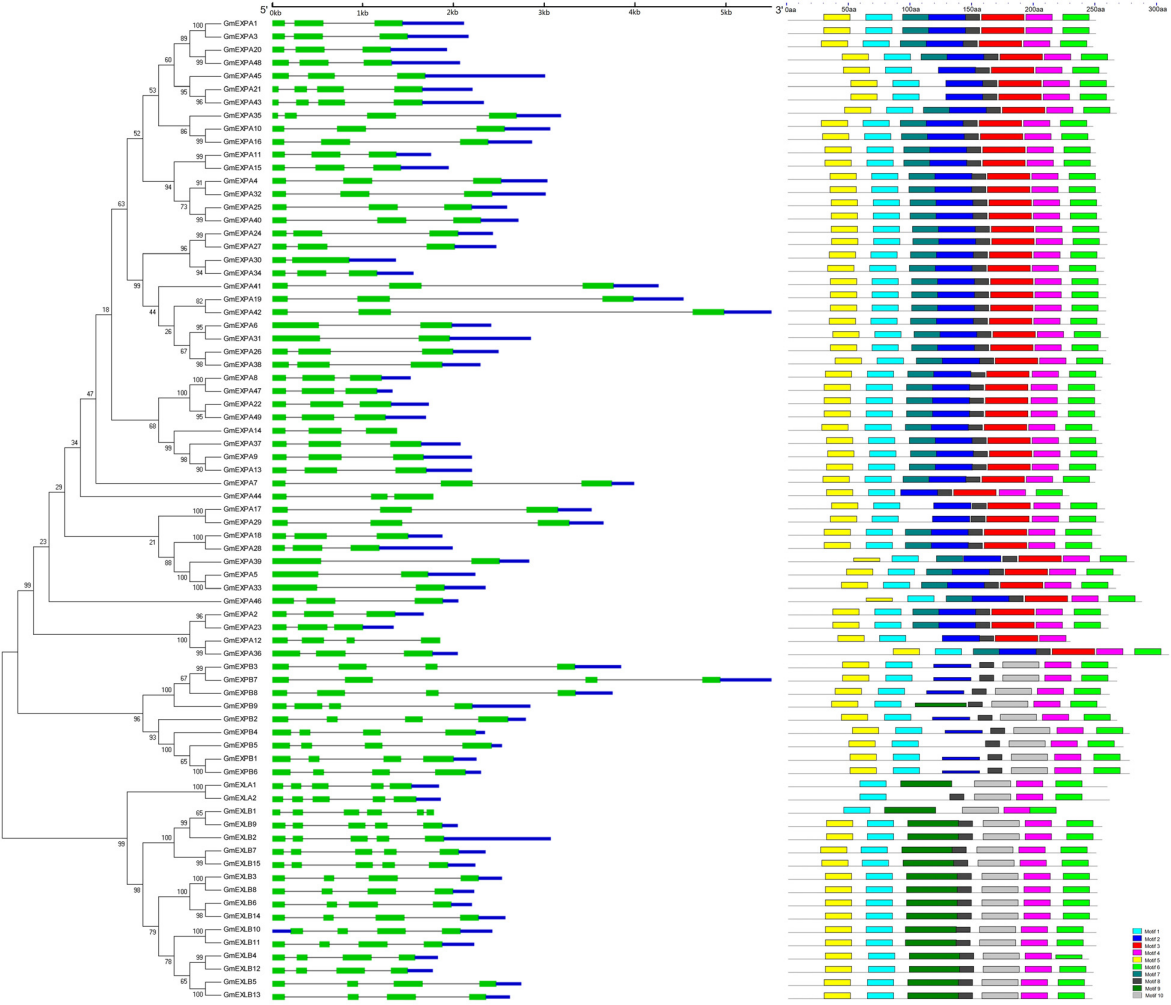
**An analytical view of the soybean expansin gene superfamily.** The following parts are shown from left to right. Protein neighbor-joining tree: The unrooted tree was constructed using MEGA v5.0. The expansin proteins are named from their gene name (see Table 1). Gene structure: The gene structure is presented by green boxes that correspond to exons, and linking black lines that correspond to introns, while the blue line refers to the 5′-UTR and 3′-UTR. Motif compositions: The colored boxes represent the motifs in the protein, a total of 10 types of motifs were found in these 75 expansin genes, as indicated in the table on the right-hand side. The scale at the top of the image may be used to estimate motif length. aa, amino acids. A detailed motif introduction is shown in Additional file 9.

We performed a multiple sequence alignment (Additional file 8) and constructed a phylogenetic tree of the 75 soybean expansin genes based on their deduced amino acid sequences (Figure 2). The expansin proteins from the same subfamily were clustered together. The phylogenetic classification was found to be consistent with the motif locations and exon-intron organizations among the four subfamilies.

As displayed schematically in Figure 2, 10 types of motif (Additional file 9) were detected. The type, order, and number of motifs were similar in proteins of the same subfamily, but differed to proteins in other subfamilies. In the EXPA subfamily, 85.7% (42 out of 49) of members shared the same eight motif components (motif 1 to 8) in the same order, which was significantly different to that of the other three subfamilies in which the members lacked motifs 3 and 7. Moreover, motif 10 was present in all genes of all subfamilies, except EXPA. Consequently, the motif distribution in EXPA was significantly different to that in the other three subfamilies, leading to the subfamilies EXPB, EXLA, and EXLB having a closer evolutionary and phylogenetic relationship. However, most expansins (77.8%; 7 of 9) in the EXPB subfamily contained motif 2, which was present in all expansins of the EXPA subfamily, but not in the EXLA and EXLB subfamilies. This finding indicates that EXPA and EXPB have a closer evolutionary and phylogenetic relationship compared to EXPA with the EXLA/EXLB subfamilies. Therefore, it indicates that the motif locations of expansins belonging to the same subfamily are conserved, whereas divergence exists among expansins from the four subfamilies.

The exon-intron organization of the expansin genes in soybean was examined by comparing the predicted coding sequences (CDS) with their corresponding genomic sequences through the online software GSDS (http://gsds.cbi.pku.edu.cn/), to obtain more insights about their possible gene structural evolution. Because an ATG sequence is located near to the first initiation codon of *GmEXLB10*, the software GSDS recognized the subsequent ATG as the initiation codon. Thus, the exon-intron organization of this gene was preceded by a short 5′-UTR, whereas in other genes it was not (Figure 2). Our results showed that genes in the same family generally have similar exon-intron structures, with the same number of exons. For example, all genes from the EXPB and EXLA subfamilies contain four exons, most genes from the EXPA subfamilies contain three exons, while the genes from EXLB families contain five exons. In turn, this finding supported the classification of the expansin genes in soybean. Moreover, this result reflects the divergence in the gene structure of the four subfamilies. In addition, variations are present in the exon-intron structure of genes from the EXPA and EXLB subfamilies, with several genes containing different numbers of exons. Most of the expansin genes in the EXPA subfamily contain three exons, while the remainder contains two or four exons. This variation might have resulted from the loss or gain of exons over a long evolutionary period. Furthermore, comparison of the exon-intron structure among genes from the four subfamilies indicated that the EXPB and EXLA subfamilies are more conserved compared to the EXPA and EXLB subfamilies.

The results of the phylogenetic and structural analysis revealed that each of the four subfamilies was conserved, and that there was also broad diversification among subfamilies. The high degree of sequence identity and similar exon-intron structures of expansin genes within each family indicates that the soybean expansin superfamily has undergone gene duplications throughout evolution. As a result, the expansin gene families contain multiple copies that might partially or completely overlap in function, with the analysis of the soybean gene expansion and expression pattern in this study supporting this hypothesis.

### Analysis of expansin gene expansion pattern

Gene duplications are considered to be one of the primary driving forces in the evolution of genomes and genetic systems [22]. Duplicated genes provide raw material for the generation of new genes, which, in turn, facilitate the generation of new functions. Segmental duplication, tandem duplication, and transposition events, such as retroposition and replicative transposition [23], are considered to represent three principal evolutionary patterns. Of these patterns, segmental and tandem duplications have been suggested to represent two of the main causes of gene family expansion in plants [24]. Segmental duplications multiple genes through polyploidy followed by chromosome rearrangements [25]. It occurs most frequently in plants because most plants are diploidized polyploids and retain numerous duplicated chromosomal blocks within their genomes [24]. Tandem duplications were characterized as multiple members of one family occurring within the same intergenic region or in neighboring intergenic regions [26]. In this study, we defined tandem duplicated genes as adjacent homologous genes on a single chromosome, with no more than one intervening gene. For this analysis, we focused on segmental and tandem duplication events. To gain a greater insight about the expansion pattern of soybean expansin genes in this huge gene family, we identified tandem duplicated clusters based on the gene locus, and searched the Plant Genome Duplication Database [27] to locate segmentally duplicated pairs. We searched for contiguous expansin genes in both the sharing and neighboring regions. We found that 11 out of 75 genes (14.7%) in this family are tandem repeats in soybean (Figure 1), indicating that tandem duplications have contributed to the expansion of this family. We also tested the hypothesis that segmental duplication events play an important role in the evolution of the expansin superfamily in soybean. We searched each soybean expansin gene in PGDD (http://chibba.agtec.uga.edu/duplication/), and found that 68% (51 of 75) of genes are involved in segmental duplication (Figure 1). Of interest, when we compared the 51 segmentally duplicated genes identified in our study with the results of Du et al. [28,29], 40 (78.4%; 40 of 51) expansin genes originated from whole genome duplications (WGDs), while the remaining 11 (21.6%; 11 of 51) expansin genes were singletons (*GmEXPA2*, *GmEXPA8*, *GmEXPA17*, *GmEXPA21*, *GmEXPA22*, *GmEXPA23*, *GmEXPA29*, *GmEXPA43*, *GmEXPA45*, *GmEXPA47*, and *GmEXPA49*). This finding indicates that the remaining 11 segmentally duplicated expansin genes might be derived from independent duplication events. Therefore, part of the expansin genes in soybean was retained after WGDs. Previous studies have suggested that the genes retained as duplicated pairs after WGD events tend to belong to specific classes, such as transcription factors and members of large multiprotein complexes [30-32], which supports the results of the present study.

In parallel, we calculated the 4DTv of these tandem-duplicated gene pairs (Table 2) using PAML v4.4. The 4DTv values ranged from 0, for recently duplicated peptides, to 0.5, for paralogs with an ancient evolutionary past. The results showed that all of the 4DTv values were around 0.2, much larger than 0. Hence, we deduced that the tandem-duplicated gene pairs may have an ancient evolutionary past. As shown on the gene map, two large tandem-duplicated gene clusters from the EXLB families are present on chromosome 5 and 17; thus, chromosome 17 is the chromosome with the highest density of expansin genes in soybean. Obviously, the duplication events, particularly tandem duplication, might result in the uneven distribution of expansin genes on chromosomes, to a certain extent. In addition, we used Ks as a proxy for time, and the conserved flanking protein coding genes to estimate the dates of the segmental duplication events. The mean Ks values and the estimated dates for all segmental duplication events corresponding to expansin genes are listed in Table 3. The segmental duplication events in soybean appear to have occurred during two relatively recent key periods, 10–25 mya and 40–65 mya, except for the independent duplication events. These inferences are consistent with the ages of the soybean genome duplication events, which occurred at approximately 59 and 13 million years ago [21]. This is compatible with our result that 40 (78.4%; 40 of 51) of the expansin genes originated from WGDs according to the data from Du et al. [28,29]. Therefore, our findings indicate that most of genes involved in segmental duplication are a result of whole genome duplication events, while the remainder may have arisen as a result of separate segmental duplication events.

**Table 2:** Genes involved in tandem duplication and their 4DTv values

**Tandem duplicated gene pairs**	**Chromosome**	**4DTv value**
*GmEXPA13 & GmEXPA14*	6	0.1209
*GmEXPB4 & GmEXPB5*	10	0.2139
*GmEXLB4 & GmEXLB5*	5	0.1747
*GmEXLB4 & GmEXLB5*	5	0.1925
*GmEXLB5 & GmEXLB6*	5	0.2218
*GmEXLB11 & GmEXLB12*	17	0.2180
*GmEXLB11 & GmEXLB12*	17	0.3312
*GmEXLB11 & GmEXLB12*	17	0.2881
*GmEXLB12 & GmEXLB13*	17	0.2881
*GmEXLB12 & GmEXLB13*	17	0.2881
*GmEXLB13 & GmEXLB14*	17	0.2411

**Table 3:** Estimates of the dates for the segmental duplication events of expanin gene superfamily in soybean

**Segment pairs**	**Number of anchors**	**Ks**	**Estimated time**
		**(mean ± s.d.)**	**(mya)**
*GmEXPA22 & GmEXPA49*	6	0.100 ± 0.012	8
*GmEXPA8 & GmEXPA47*	15	0.119 ± 0.054	10
*GmEXPA4 & GmEXPA32*	10	0.145 ± 0.024	12
*GmEXPA30 & GmEXPA34*	20	0.149 ± 0.054	12
*GmEXPA24 & GmEXPA27*	18	0.157 ± 0.118	13
*GmEXPA11 & GmEXPA15*	15	0.175 ± 0.141	14
*GmEXPA6 & GmEXPA31*	13	0.177 ± 0.213	14
*GmEXPA9 & GmEXPA13*	19	0.188 ± 0.172	15
*GmEXPA21 & GmEXPA43*	17	0.202 ± 0.166	17
*GmEXPA12 & GmEXPA36*	16	0.205 ± 0.096	17
*GmEXPA2 & GmEXPA23*	20	0.239 ± 0.253	20
*GmEXPA26 & GmEXPA38*	14	0.254 ± 0.219	21
*GmEXPA1 & GmEXPA3*	5	0.270 ± 0.132	22
*GmEXPA18 & GmEXPA28*	5	0.296 ± 0.266	24
*GmEXPA17 & GmEXPA29*	4	0.300 ± 0.179	25
*GmEXPA8 & GmEXPA49*	4	0.453 ± 0.085	37
*GmEXPA16 & GmEXPA35*	5	0.477 ± 0.346	39
*GmEXPA47 & GmEXPA49*	4	0.515 ± 0.139	42
*GmEXPA22 & GmEXPA47*	8	0.531 ± 0.118	44
*GmEXPA8 & GmEXPA22*	8	0.539 ± 0.132	44
*GmEXPA24 & GmEXPA34*	9	0.598 ± 0.189	49
*GmEXPA27 & GmEXPA34*	9	0.613 ± 0.180	50
*GmEXPA24 & GmEXPA30*	11	0.617 ± 0.158	51
*GmEXPA27 & GmEXPA30*	11	0.626 ± 0.155	51
*GmEXPA6 & GmEXPA38*	4	0.633 ± 0.257	52
*GmEXPA2 & GmEXPA36*	6	0.650 ± 0.158	53
*GmEXPA6 & GmEXPA26*	4	0.650 ± 0.177	53
*GmEXPA26 & GmEXPA31*	4	0.680 ± 0.163	56
*GmEXPA23 & GmEXPA36*	5	0.685 ± 0.135	56
*GmEXPA2 & GmEXPA12*	7	0.708 ± 0.099	58
*GmEXPA13 & GmEXPA37*	7	0.710 ± 0.102	58
*GmEXPA9 & GmEXPA37*	7	0.763 ± 0.112	63
*GmEXPA12 & GmEXPA23*	6	0.768 ± 0.078	63
*GmEXPA21 & GmEXPA45*	3	0.790 ± 0.207	65
*GmEXPA43 & GmEXPA45*	3	0.817 ± 0.266	67
*GmEXPB8 & GmEXPB9*	16	0.169 ± 0.077	14
*GmEXPB3 & GmEXPB7*	6	0.397 ± 0.277	33
*GmEXLA1 & GmEXLA2*	23	0.167 ± 0.072	14
*GmEXLB3 & GmEXLB8*	19	0.176 ± 0.117	14
*GmEXLB5 & GmEXLB12*	8	0.191 ± 0.165	16
*GmEXLB7 & GmEXLB15*	7	0.202 ± 0.149	17
*GmEXLB6 & GmEXLB14*	8	0.211 ± 0.179	17
*GmEXLB2 & GmEXLB9*	15	0.236 ± 0.216	19
*GmEXLB2 & GmEXLB15*	3	0.447 ± 0.029	37
*GmEXLB9 & GmEXLB15*	3	0.503 ± 0.068	41
*GmEXLB3 & GmEXLB6*	3	0.513 ± 0.093	42
*GmEXLB3 & GmEXLB12*	6	0.630 ± 0.117	52
*GmEXLB4 & GmEXLB8*	4	0.685 ± 0.227	56

Overall, these results indicate that the expansin gene superfamily has expanded by both segmental and tandem duplication, particularly segmental duplication. Furthermore, most of the genes involved in segmental duplication were retained after WGDs.

### Expression analysis of expansin gene superfamily in soybean

**Figure 3:**
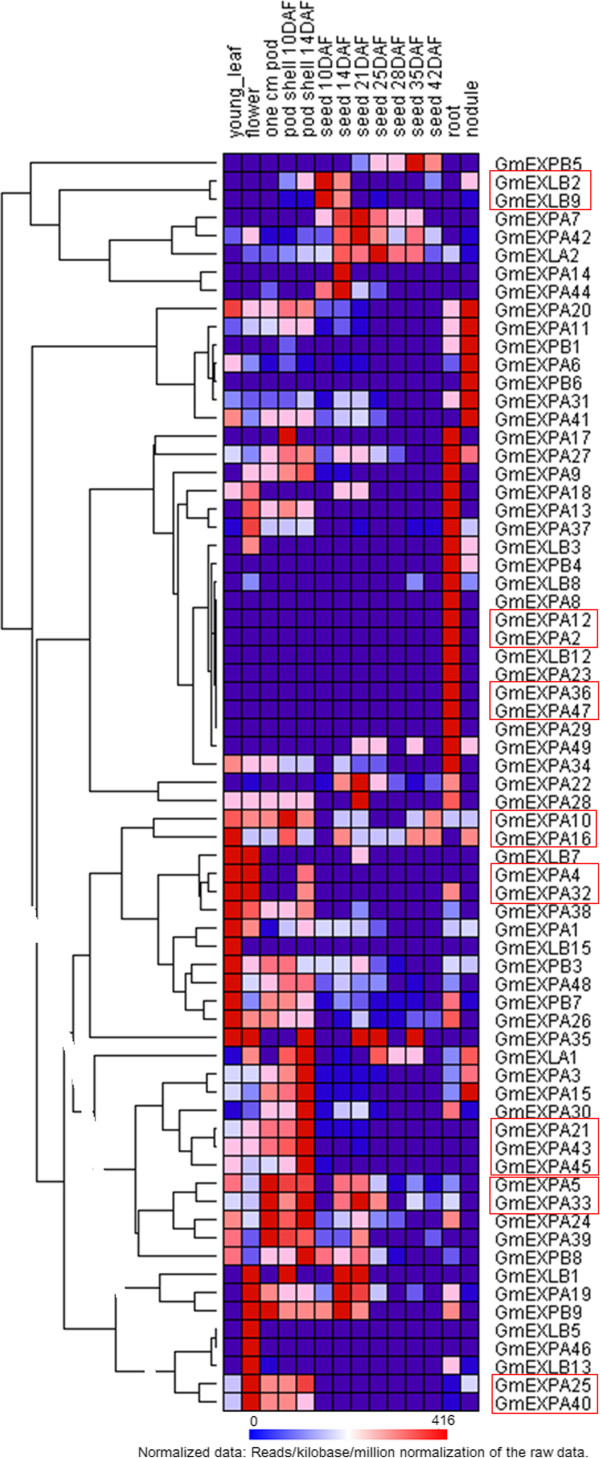
**Expression profiles of the 75 soybean expansin genes.** The hierarchical cluster color code: the largest values are displayed as the reddest (hot), the smallest values are displayed as the bluest (cool), and the intermediate values are a lighter color of either blue or red. Raw data were normalized by the following equation: reads/kilobase/million. Pearson correlation clustering was used to group the developmentally regulated genes. Six genes were excluded from the analysis because they were not expressed in an organ or a period. The red outlined boxes represent the small phylogenetic clades that had a largely similar transcript abundance profile.

The recently developed RNA-Seq web-based tools, which include gene expression data across multiple tissues and organs, allow for characterization and comparison of the gene transcriptome atlas in soybean. Consequently, distinct transcription abundance patterns are readily identifiable in the RNA-Seq atlas dataset for soybean expansin genes. The RNA-Seq atlas data of soybean expansin genes (Additional file 10) were downloaded from Soybase (http://soybase.org/soyseq/). However, six expansin genes (*GmEXPB2*, *GmEXLB4*, *GmEXLB6*, *GmEXLB14*, *GmEXLB10*, and *GmEXLB11*) lacked RNA-Seq atlas data, which might indicate that these genes are pseudogenes, or are only expressed at specific developmental stages or under special conditions. The RNA-Seq atlas analysis indicated that many of the soybean expansin genes exhibited low transcript abundance levels. We observed that the accumulation of expansin gene transcripts was associated with different tissues, and that the expression patterns differed among each expansin gene member (Figure 3). In soybean, 31% (23 of 75) of the analyzed expansins were constitutively expressed in all of the seven tissue types examined. This finding indicates that expansins are involved in multiple processes during the development of soybean. In contrast, most soybean expansins exhibited preferential expression. The RNA-Seq atlas data revealed that the majority (72%; 54 of 75) of soybean expansins exhibit transcript abundance profiles with marked peaks in only a single tissue type. This result indicates that these expansins function as cell wall loosening proteins, and are limited to discrete cells or organs. Approximately 25% (total n =75 ), 20%, 13%, 11%, 9%, and 7% soybean expansins exhibited the highest transcript accumulation level in root tissue, seed tissue, pod shell tissue, leaf tissue, nodule tissue, and flower tissue, respectively. The first reported root-specific soybean expansin gene [33] has a high expression level in the root, and plays an important role in the root of soybean. According to the gene loci, it only corresponds to *GmEXPA37* (Glyma17g37990). As shown in Figure 3, *GmEXPA37*has a marked peak in the transcript abundance profile of root tissue only, which is consistent with previous research [33]. According to the Libault Atlas [34] (Additional file 11), *GmEXPA37* tends to be expressed in root hairs; hence, it might contribute to the development of root hairs. The wide expression of these genes indicates that expansin genes from soybean are involved in the development of all organs and tissues under normal conditions. Although expansin genes might have general, overlapping expression in some instances, in other cases, expression might be highly specific, and limited to a single organ or cell type. Some expansins were only expressed in a single tissue: seven genes (*GmEXPA12*, *GmEXPA8*, *GmEXPA2*, *GmEXLB12*, *GmEXPA23*, *GmEXPA29*, *GmEXPA36*, and *GmEXPA47*) were only expressed in root; three genes (*GmEXPA7*, *GmEXPA14,* and *GmEXPB5*) were only expressed in the seed; two genes (*GmEXLB5* and *GmEXPA46*) were only expressed in the flower; one gene (*GmEXPB6*) was only expressed in the nodule; and one gene (*GmEXLB15*) was only expressed in the leaf. Our analysis indicated that these genes might be tissue-specific or, at least, preferentially expressed. Interestingly, these results showed that more genes of the expansin gene superfamily might be specifically or preferentially expressed in the root. Another heatmap (Additional file 11) based on the Libault Atlas provided more information about the genes that were preferentially expressed in roots. The Libault atlas focus on the below ground tissues and provide more information about the genes highly expressed in the underground tissues, especially in root, root hair, root tip.

In addition, expansin genes that were clustered in branches in the heatmap exhibited similar transcript abundance profiles. However, most of these genes were not clustered in the phylogenetic tree and were relatively phylogenetically distinct. Only several small phylogenetic clades had largely similar transcript abundance profiles, and were marked on the heatmap in red outlined boxes (Figure 3). Soybean expansins that have high sequence similarity and share expression profiles represent good candidates for the evaluation of gene functions in soybean. Therefore, genes in the red outlined boxes may have a similar function in the same tissues. For example, *GmEXPA2* and *GmEXPA12*, which were clustered in the phylogenetic tree with high sequence similarity only expressed in the root tissue, which indicates that both genes may have the same function in the root tissue.

The transcriptome atlas indicated that all four subfamilies of the soybean expansin superfamily were differentially expressed, which may be associated with the divergence of the promoter regions of the expansin genes. Promoters in the upstream region of genes play key roles in conferring developmental and/or the environmental regulation of gene expression [35]. Thus, profiles of *cis*-acting elements may provide useful information about the regulatory mechanism of gene expression. A computational tool, PlantCARE [36], was used to identify *cis*-acting elements in the 1500-bp DNA sequence upstream of the translation initiation codon of expansin genes in soybean. Four types of *cis*-acting element were found to be significantly abundant in the promoter region of the soybean expansin gene superfamily (Additional file 12). The first type of *cis*-acting element enriched in the promoter region is the light-responsive element, which includes the G-box [37,38], Box 4 [39], and Box I [40]. The G-box appears to be the most abundant light-responsive element in soybean expansin genes, with a mean number of 1.386 copies, while the G-box is less abundant in EXLB (mean number of 0.8000 copies) compared to the other three subfamilies. Another class of *cis-*acting elements enriched in the promoter region of expansin genes is the plant hormone-responsive elements, including the TCA-element [41], TGA-element [42], and GARE-motif [43]. The salicylic acid-responsive TCA-element appears to be the most abundant hormone-related *cis*-acting element in soybean expansin genes, indicating that salicylic acid regulates the expression of some soybean expansin genes. The abundance of the TGA-element and GARE-motif in soybean expansin genes indicates that auxin and gibberellin also play roles in regulating soybean expansin gene expression. Other elements are also related to auxin- or gibberellin-responsiveness, such as AuxRR-core [44], TGA-box [45], P-box [46], and TATC-box [47]. These results are consistent with previous studies, which reported that some expansins are regulated by auxin [48,49] and gibberellin [50,51]. The third most abundant *cis*-acting element class contains elements that respond to external environment stresses. We observed that most soybean expansin genes appeared to contain ARE [52], MBS [53], HSE [54], and TC-rich elements [52]. ARE is an element involved in anaerobic induction; hence, we speculated that the anaerobic regulation of expansin expression could be tissue or developmental stage depend. The drought-responsive element MBS is also abundant in the promoter region. With few exceptions, expansin genes contain at least one copy of this element (Additional file 12). These results are consistent with the fact that expansin activities have been found to be influenced by various abiotic stressors, including drought [55,56] and flooding [57-61]. Circadian elements, which are involved in circadian control [62], comprise the fourth class of *cis*-acting element that was abundantly found in the promoter region of soybean expansin genes. PlantCARE analysis showed that soybean expansin genes contain circadian elements, potentially indicating that expansin has a distinct diurnal expression pattern [63]. Promoter analysis demonstrated the presence of a diversity of *cis*-acting elements in the upstream regions of the soybean expansin gene superfamily. This finding provides further support for the various functional roles of expansins in a wide range of developmental processes related to cell wall modification.

These results indicate that the 75 expansin genes in soybean display differential expression in the four subfamilies, either in the abundance of their transcripts or in their expression patterns under normal growth conditions.

### Functional divergence analysis of soybean expansin proteins

Functional divergence among the subfamilies of the soybean expansin superfamily was inferred by posterior analysis using the program DIVERGE v2.0. The posterior probability (Qk) of divergence at each site was calculated to predict the location of certain critical amino acid sites (CAASs) [64] that are highly relevant to functional divergence. In our study, two types of functional divergenence were estimated. Type-I functional divergence refers to the evolutionary process resulting in a site-specific shift in the evolutionary rate after gene duplication, whereas Type-II functional divergence refers to the site-specific amino acid physiochemical property shift. These methods have been extensively applied to the research of various gene families, as they are not sensitive to the saturation of synonymous sites [64-66]. The estimate was based on the neighbor-joining tree constructed from all of the protein sequences of the 75 soybean expansin genes. In comparison, the subfamily EXLA, which contains only two members was excluded, because groups with less than four sequences cannot be analyzed using this method. Pairwise comparisons of paralogous expansin genes from the remaining three subfamilies were carried out, and the rate of amino acid evolution at each sequence position was estimated. Our results (Table 4) indicate that the coefficients of Type-I functional divergence (θI) among the three expansin subfamilies were strongly statistically significant (p < 0.01), with the θI values ranging from 0.498 to 0.783. Hence, significant site-specific changes altered the selective constraints on expansin members of the superfamily, leading to subgroup-specific functional evolution after diversification. Type-II functional divergence (θII) between the subfamilies (EXPA/EXLB) was evident with an θII value of 0.136 (p < 0.05), which is suggestive of a radical shift in amino acid properties. The coefficients of type II functional divergence θ between EXPA/EXPB and EXPB/EXLB were not that evident, with θII values smaller than 0 being obtained, but with high standard errors. Hence, the relative importance of Type-I and Type-II functional divergence appears to be different regarding the functional divergence of subfamilies of the soybean expansin superfamily.

**Table 4:** Functional divergence between subfamilies of the expansin gene superfamily in soybean

**Group I**	**Group II**	**Type-I**	**LRT**	**Qk > 0.95**	**Critical amino acid sites**	**Type-II**	**Qk > 0.95**	**Critical amino acid sites**
		**θ**_**I**_ **± s.e.**				**θ**_**II**_ **± s.e.**		
EXPA	EXPB	0.498 ± 0.079	39.742	3	84C,**145 V**,172 L	-0.023 ± 0.259	15	62 T,65 L,103 F,**104C**,121P,122 M,141G,
								143 V,160 F,176 V,177G,190*S,**191R**,207S
EXPA	EXLB	0.783 ± 0.082	91.136	17	**45G**,**54Y**,**61 N**,84C,**102 N**,**104C**,	0.136 ± 0.278	53	18A,**45G**,**54Y**,56*Q,60 T,**61 N**,65 L,67 T,69 L,
					161 T,**165H**,167Y,172*L,**176 V**,			72 N,75S,76C,82I,**102 N**,**104C**,106P,107 N,
					**181D**,184*G,**185 V**,**191R**,**201 W**,			120P,125 F,126D,127 L,133*L,137Q,138Y,
					202G			**145 V**,147Y,154R,155R,160 F,162I,**165H**,
								168 F,170 L,175 N,**176 V**,180G,**181D**,**185 V**,
								187I,189G,**191R**,192*T,196*P,199R,**201 W**,
								204 N,205 W,207S,208 N,209 N,210Y,213G
EXPB	EXLB	0.572 ± 0.141	14.448	0	none	-0.081 ± 0.298	13	**54Y**,63A,67 T,103 F,106P,121P,125 F,126D,
								134R,137R,140A,175 N

Note: θI and θII, the coefficients of Type-I and Type-II functional divergence;

LRT, Likelihood Ratio Statistic;

Qk, posterior probability;

*Sites also responsible for the positive selection;

Sites in bold means they are responsible for both type-I and type-II functional divergence.

Furthermore, we predicted that some critical amino acid residues are responsible for functional divergence, with suitable cut-off values being derived from the Qk of each comparison. Given that too many functional divergence-related residues (data not shown) were identified by DIVERGE2 when the empirically Qk value 0.8 was used as a cutoff value, we used Qk > 0.95 to predict CAASs to exclude other sites for further analysis. As a result, a total of 19 CAASs were predicted through type-I functional divergence analysis, whereas 63 amino acid sites with fairly high probability (Qk > 0.95) were identified through type-II functional divergence analysis, which is indicative of a radical shift in evolution rate and amino acid properties to some extent. Furthermore, 12 amino acids are crucial for both the type-I and the type-II functional divergence, indicating that shifts in evolutionary rates and altered amino acid physicochemical properties co-occurred at the these amino acid sites. Hence, these sites probably played important roles in functional divergence during the evolutionary process. In addition, we also noticed that the number of predicted sites (Table 4) within each pair differs between type-I and type-II functional divergence; namely, more CAASs were identified by type-II functional divergence within each subfamily pair. Hence, the functional divergence between the genes of the two groups is mainly attributed to rapid changes in amino acid physiochemical properties, followed by the shift in the evolutionary rate.

Besides, in contrast with EXPA/EXPB and EXPB/EXLB, EXPA/EXLB had relatively larger coefficients of functional divergence (θI & θII) and much more sites that were related to functional divergence. Hence, the functional divergence that exists between EXPA and EXLB is more significant compared with that present in EXPA/EXPB and EXPB/EXLB, although no biological or biochemical function has yet been established for any members of EXLB [8]. In addition, we also deduced that a lesser degree of functional divergence occurred within EXPA/EXPB and EXPB/EXLB based on the coefficients of functional divergence and the number of identified CAASs. Hence, EXPB and EXLB have a much closer phylogenetic relationship compared with EXPA and EXLB, which was also indicated by the motif analysis. The motif analysis showed that the EXPA subfamily has a clearly different motif organization compared to the other two subfamilies, whereas the EXPB and EXLB subfamilies shared similar types and numbers of motifs.

### Positive selection analysis

To test the hypothesis of positive selection in soybean expansin genes, we used the site model and the branch site model in the CODEML program of the PAML v4.4 software package [67]. The substitution rate ratios of non-synonymous (dN or Ka) versus synonymous (dS or Ks) mutations (dN/dS or ω) were calculated. The Ka/Ks ratio should be 1 for genes subject to neutral selection, <1 for genes subject to negative selection, and >1 for genes subject to positive selection [68]. In the site model, codon site models M0, M3, M7, and M8 were implemented, using likelihood ratio tests to test whether variable ω (dN/dS) ratios were present at the amino acid sites. M0 is the one-ratio model that assumes one ω ratio at all sites. In the discrete model (M3), the probabilities (p0, p1, and p2) of each site were submitted to purifying, neutral, and positive selection, respectively, and their corresponding ω ratios (ω0, ω1, and ω2) were inferred from the data. The Beta model (M7) is a null test for positive selection, assuming a Beta distribution with ω between 0 and 1. Finally, the Beta & ω model (M8) add one extra class with the same ratio ω1 [69]. In our study, two pairs of models (M0/M3 and M7/M8) were selected and compared (Table 5). First, models M0 and M3 were compared, using a test for heterogeneity between codon sites in the dN/dS ratio value, in which twice the log likelihood difference, 2Δℓ = 560, would indicate a strongly statistically significant result (p < 0.01), reflecting large selective pressure on the soybean expansin superfamily; namely, soybean expansin has undergone strong positive selection. The comparison of M3 versus M0 revealed that none of the codon sites appeared to be under the influence of positive selection (ω > 1). In contrast, the comparison of M7 (beta) and M8 (beta + ω > 1), which is considered to be the most stringent test of positive selection [70], indicated that ~0.001% codons fell within an estimated ω value of 2.02644 (which is suggestive of positive selection). On the basis of the Bayesian posterior probabilities, 14 codon site candidates (42G, 43 T, 123H, 146S, 153R, 166S, 172 L, 184G, 186A, 190S, 195 M, 196P, 198S, and 203Q) for positive selection were identified from the M8 models. Of these sites, eight positive selection sites were at the 0.01 significance level, while the remainder was at the 0.05 significance level. Four amino acid residues (172 L, 184G, 190S, and 196P) that were identified in the site-model were also responsible for functional divergence; namely, 172 L and 184G were responsible for type-I functional divergence, while 190S and 196P were responsible for type-II functional divergence.

**Table 5:** Tests for positive selection among codons of expansin genes using site models

**Models**	***p***^***a***^	**Estimates of parameters**	**InL**	**2⊿l**	**Positively selected sites**^**b**^
M0	1	ω = 0.133	-14554.8		None
(one-ratio)					
M3	5	p_0_ = 0.22607 p_1_ = 0.55054 p_2_ = 0.22339	-14274.8	560(M3 *vs*M0)	None
(discrete)		ω_1_ = 0.02570 ω_2_ = 0.11359 ω_3_ = 0.33505			
M7	2	p = 0.99176 q = 5.71801	-14266.9		Not allowed
(beta)					
M8	4	p0 = 0.99999 p = 0.61117 q = 1.88462	-16630.6	4727.4(M8 *vs*M7)	**42G,43T,123H,146S,153R,**166S,172L*,
(beta&ɯ)		(p1 = 0.00001) ω = 2.02644			184G*,186A,190S*,195 M,196P*,198S,203Q

Note: ^a^Number of parameters in the ω distribution.

^b^Positive-selection sites are inferred at posterior probabilities > 95% with those reaching 99% shown in bold.

*Sites were also found to be implicated in the functional divergence.

In the branch site model, ω is allowed to vary both among sites in the protein and across branches on the tree, with the aim of detecting positive selection that only affects a few sites along particular lineages [71]. The branches being tested for positive selection are referred to as the foreground branches, while the remaining branches on the tree are referred to as background branches. The BEB method was implemented to calculate posterior probabilities (Qks) for site classes if the LRT indicates the presence of codons under positive selection on the foreground branch [67]. Each soybean expansin subfamily was selected as a foreground branch, to test for positive selection. The results (Table 6) show that divergent positive selection was detected among the four subfamilies. When EXPB, EXLA, or EXLB were selected as the foreground branch, the foreground ω values were fairly large, and nearly all codon site candidates were identified; however, none of the codons had a posterior probability higher than 0.95, except for 192 T, which had a posterior probability of 0.984 when EXLB was chosen as the foreground branch. No sites with posterior probabilities higher than 0.95 were found when the EXPB or EXLA subfamily was chosen as the foreground branch. However, positive selection often acts on a few sites and in a short period of evolutionary time; hence, the signal may be swamped by widespread negative selection [72]. In contrast, when EXPA was chosen as the forebranch, the foreground ω value (1.32036) was much lower, and a total of 10 sites (56Q, 133 L, 166S, 169 N, 186A, 172 L, 174 T, 190S, 198S, and 203Q) with posterior probabilities higher than 0.95 being identified.

**Table 6:** Parameters estimation and likelihood ratio tests for the branch-site models

**Cluster**	**Site class**	**Proportion**	**Backgroudɯ**	**Foregroudɯ**	**Positive selected sites**^**a**^
EXPA	0	0.69757	0.12227	0.12227	56Q*,133 L*,166S,**169N**,186A,172 L*,**174 T**,190S*,198S,203Q
	1	0.07472	1	1	
	2a	0.20568	0.12227	1.32036	
	2b	0.02203	1	1.32036	
EXPB	0	0.70168	0.12941	0.12941	none
	1	0.08281	1	1	
	2a	0.19276	0.12941	999	
	2b	0.02275	1	999	
EXLA	0	0.43399	0.12875	0.12875	none
	1	0.05093	1	1	
	2a	0.46097	0.12875	999	
	2b	0.0541	1	999	
EXLB	0	0.49513	0.12363	0.12363	192 T*
	1	0.05939	1	1	
	2a	0.39777	0.12363	999	
	2b	0.04771	1	999	

Note: ^a^Positive-selection sites are inferred at posterior probabilities > 95% with those reaching 99% shown in bold.

*Sites were also found to be implicated in the functional divergence.

These results indicate divergent selective constraints on the four subfamilies. The EXPB and EXLA subfamilies are considerably more conserved compared to the EXPA and EXLB subfamilies. Furthermore, the EXPA subfamily might have been subject to the strongest positive selection among the four subfamilies, as the most highly significant positive sites were detected in this subfamily.

## Discussion

### Origin of the soybean expansin gene superfamily

Recent research studies have assumed that 70% ~ 80% of angiosperms have undergone duplication events [73-76]. For example, 90% and 62% of *Arabidopsis thaliana* and *Oryza sativa* loci have undergone duplication events [22]. As an ancient polyploid, soybean has a highly duplicated genome, with nearly 75% of the genes present occurring in multiple copies [21]. The current investigation revealed the duplication pattern of the soybean expansin gene family. Eleven genes were identified as tandem repeats, indicating that tandem duplication has also contributed to the expansion of the soybean expansin gene superfamily. In addition, 51 genes were found to have evolved from segmental duplication, indicating that segmental duplication probably played a pivotal role in expansin gene expansion in the soybean genome. The genome sequencing results revealed that whole genome duplications (WGD) in soybean occurred at approximately 59 and 13 million years ago (MYA), which is consistent with results of the present study. We inferred that expansion of the expansin gene family occurred along with WGD events, and that these genes were retained during evolution. Previous research has indicated that rapid functional divergence and the biased expression of duplicated genes appear to be major factors promoting their retention in the genome [77-81]. In our study, significant functional divergence was identified among the four subfamilies, with duplicated genes exhibiting diverse expression. For instance, in one duplicated gene pair, *GmEXPA30* &*GmEXPA34*, the two genes were retained after genome duplication events, with only *GmEXPA30* being expressed in the leaf, indicating biased expression. Similar cases have also been observed in other segmentally duplicated gene pairs, such as *GmEXPA4* &*GmEXPA32*, *GmEXPA6 *&*GmEXPA31*, and *GmEXPA18* &*GmEXPA28*. These results further verified our hypothesis that most of the segmentally duplicated soybean expansin genes have been retained from genome duplication events. Analysis of the expansion pattern of the expansin gene superfamily revealed that the soybean genome had undergone large-scale duplication. Both segmental and tandem duplication are important contributors to the expansion of the expansin gene superfamily.

We also analyzed the expansion pattern of the expansin superfamily in *Arabidopsis* (Additional file 13) and rice (Additional file 14). The results of the present study showed that 50% (18 of 36) of genes were involved in segmental duplication, while 27.8% (10 of 36) of genes were involved in tandem duplication in *Arabidopsis*. In comparison, 27.6% (16 of 58) of genes were involved in segmental duplication and 55.2% (32 of 58) of genes were involved in tandem duplication in rice. In soybean, 68% (51 of 75) of genes were involved in segmental duplication and 14.7% (11 of 75) of genes were involved in tandem duplication. Hence, we observed that both segmental and tandem duplication have played significant roles in the expansion of the expansin superfamily in soybean, *Arabidopsis*, and rice. Previous studies have revealed that genes encoding transcription factors and ribosomal components are significantly over-retained following tetraploidy [82]. However, genes influencing the stress response have an elevated probability of retention following tandem duplication [83]. Expansin genes are associated with cell wall enlargement. However, while these genes are not transcription factors, ribosomal components, or genes that influence stress response, they have expanded through both tandem and segmental duplication, instead of just one form of duplication or the other. More intriguingly, we also noticed that the three species showed species-specific expansion patterns. For instance, segmental duplication seemed to be the predominant form of expansion of the expansin gene superfamilies of the two dicots, *Arabidopsis* and soybean. In contrast, tandem duplication seemed to be the predominant form of the expansion way for the expansin gene family of the monocot, rice.

### The much larger family size of EXPB in rice and EXLB in soybean

Previous studies have shown that β-expansin genes are particularly numerous and abundantly expressed in grasses, but are also found in reduced numbers in dicots [84]. Our results comparing the size of the expansin gene family in soybean, *Arabidopsis*, and rice are consistent with these previous studies. The EXPB family in rice is much larger compared to that of soybean and *Arabidopsis*. We also found that the EXLB family is much larger in soybean compared with *Arabidopsis* and rice. However, the EXPA family had the largest size in all three species. Previous research has shown that major variations in family size and the distribution of most gene families are affected by tandem duplications and segmental duplications [24]. Consequently, we compared the duplication events of the four subfamilies in the three species (Table 7). Major variation was exhibited among the subfamilies and species. The much larger size of the EXPB family in rice might have been caused by this family expanding at a different rate compared with that in the other two species. All of the genes of the EXPB family in rice were involved in segmental or tandem duplication; however, only four were involved in both segmental and tandem duplication, whereas only part of the genes of the EXPB families in soybean and *Arabidopsis* were involved in duplication events. Alternatively, from the perspective of adaptiveness, more genes of the EXPB subfamily in rice might be retained after duplication events, whereas the EXPB subfamily in soybean and *Arabidopsis* were subject to large-scale gene loss, leading to fewer EXPB genes being retained. Thus, the higher degree of expansion and retention of the EXPB family in rice caused it to become much larger. Similarly, the higher degree of expansion and retention might also explain the much larger size of the EXLB subfamily in soybean. Our results indicated that tandem duplication was the predominant contributor to the expansion of the soybean EXLA subfamily and 74% genes in EXLB that were involved in tandem duplication, and may be retained over a long evolutionary period. However, the genes of the EXLB subfamilies in *Arabidopsis* and rice were not involved in segmental or tandem duplication events. Therefore, both segmental and tandem duplication events contributed to the ever-expanding EXLB subfamily in soybean.

**Table 7:** **Duplication events of the four expansin subfamilies in Soybean, *Arabidopsis *** **andrice**

	**Segmental duplication**				**Tandem duplication**			
	**EXPA**	**EXPB**	**EXLA**	**EXLB**	**EXPA**	**EXPB**	**EXLA**	**EXLB**
Soybean	67.3% (33 of 49)	44.4% (4 of 9)	100% (2 of 2)	73.3% (11 of 15)	4.1% (2 of 49)	22.2% (2 of 9)	0% (0 of 2)	46.7% (7 of 15)
*Arabidopsis*	61.5% (16 of 26)	33.3% (2 of 6)	0% (0 of 3)	0% (0 of 1)	23.1% (6 of 26)	33.3% (2 of 6)	66.7% (2 of 3)	0% (0 of 1)
Rice	14.7% (5 of 34)	47.4% (9 of 19)	50% (2 of 4)	0% (0 of 1)	52.9% (18 of 34)	73.7% (14 of 19)	0% (0 of 4)	0% (0 of 1)

Recent research has shown that Zea m 1 (EXPB1 from maize) and orthologous group-1 pollen allergens in other grasses are highly abundant in pollen. These genes may induce extension only in grass cell walls, but are not effective on the walls of dicots, aiding the penetration of the pollen tube through the stigma and style by softening the maternal cell walls [9,84]. Moreover, β-expansin genes are particularly numerous and abundantly expressed in grasses [84]. In this study, we deduced that the size of the rice EXPB subfamily has increased to adapt to specific functional needs during the long evolutionary timeframe. Alternatively, more genes of the rice EXPB subfamily might have been subjected to a higher degree of post-duplication retention for important functions in rice development. In comparison, the genes of the EXPB subfamily of the other two species might have undergone large-scale gene loss during evolution. The even larger size of the EXLB subfamily in soybean might also reflect adaptations to certain functions or environments. Hence, the EXLB members might have a special function in soybean development; however, experimental evidence has yet to establish their activity in the cell wall [8].

### Functional divergence and positive selection analysis

Gene duplications are considered to be one of the primary driving forces in the evolution of genomes and genetic systems [22]. Typically, an amino acid residue is highly conserved in one duplicate gene, but highly variable in the other one [85]. Amino acid site mutation is frequent, with the accumulation of mutations potentially contributing to the functional divergence of duplicated genes [30,80,86,87]. Through the functional divergence analysis, critical amino acid sites (Table 4) were detected. These sites are major contributors to the functional divergence among the four soybean subfamilies. Rapid functional divergence and the biased expression of duplicated genes is expected to promote retention of the gene of the two homologs, or homoeologs derived from WGD [77-81]. In our study, the expansin gene superfamily has undergone large-scale gene duplication, with many genes being retained after WGD events. Mutations of duplicated genes, and the subsequent selection constraints on them, are expected to lead to functional divergence. At the molecular level, amino acid changes that result in reduced fitness are removed by negative selection, whereas changes that increase fitness are retained by positive selection [88]. Through positive selection analysis, amino acid sites that have undergone strong positive selection (Tables 5, 6) were also identified. Finally, we identified seven sites (56Q, 133 L, 172 L, 184G, 190S, 192 T, and 196P) that were responsible for both functional divergence and positive selection, indicating that these sites were important in the evolutionary history of the expansin gene superfamily in soybean.

**Figure 4:**
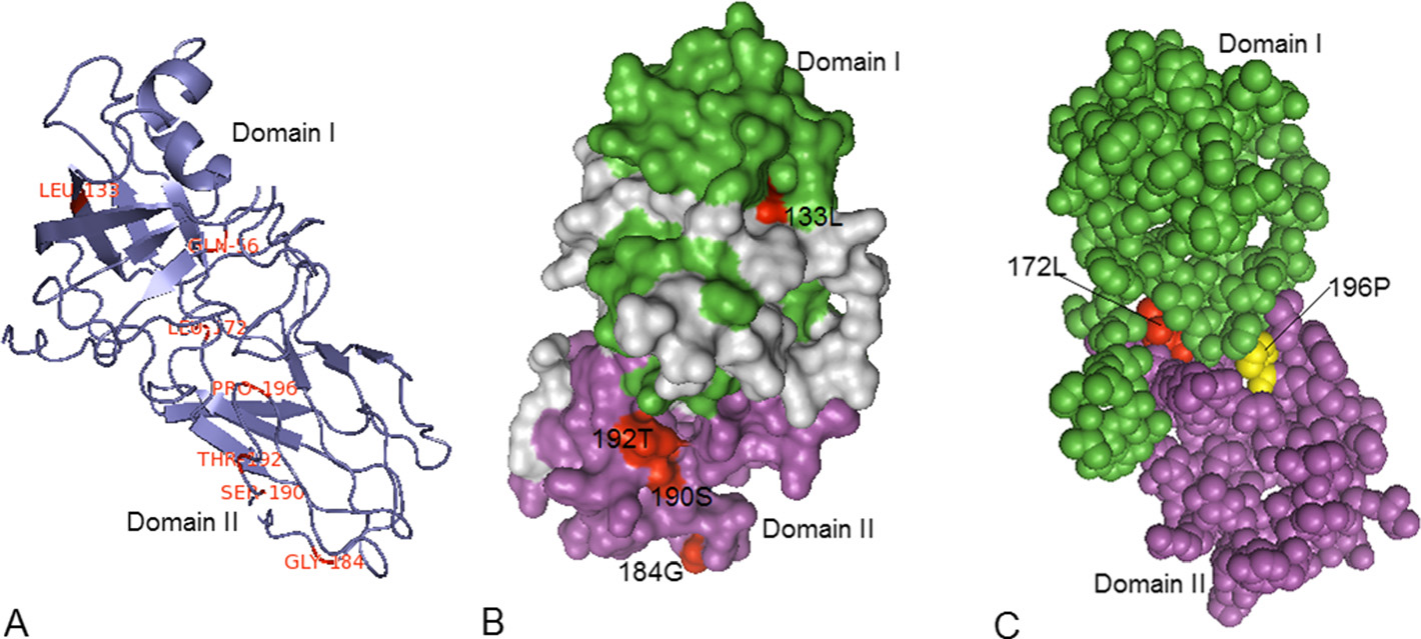
**Model building of the 3D structure of the soybean expansin protein (GmEXPA1) based on its similarity to the Zea m 1 (Protein Data Bank [PDB] code: 2HCZ).** Seven critical amino acid sites responsible for both functional divergence and positive selection are shown to varying degrees in **(A)**, **(B)**, and **(C)**. The figure was produced using the Swiss-model and pyMOL programs. **(A)** The overall view of the seven critical amino acid sites on the 3D structure. The seven sites are labeled in red. **(B)** View of critical amino acid sites on the surface of GmEXPA1. Four amino acid sites responsible for both functional divergence and positive selection located on the surface of the molecule are colored red. Of these amino acid sites, 190S and 192 T may be critical for polysaccharide binding of domain II. The gray area represents the N and C termini. **(C)** Critical amino acid sites related to the contact of the two domains. Only the two domains are shown to better exhibit the two residues, 172 L and 196P, which may be related to the contact of the two domains.

We used the Swiss-model [89-91] to model the three-dimensional structure of *GmEXPA1* through homology-modeling, and labeled the seven critical amino acid sites on it. The 3D structure shows that 172 L and 196P are located on the surface where two domains come into contact (Figure 4). Compared with the crystal structure of Zea m 1 [20], we inferred that 172 L may be involved in the contact of the two domains of the expansin protein, because it corresponds to 164 L of Zea m 1, which is located in a hydrophobic patch associated with the contact of the two domains, based on Clustal comparison of the two sequences. Consequently, we inferred that the non-polar residue 196P might also be involved in the contact of the two domains. It has been speculated that domain II is a polysaccharide-binding domain, based on the presence of conserved aromatic and polar residues on the surface of the protein [18]. Interestingly, four critical amino acid sites (133 L, 184G, 190S, and 192 T) are located on the surface of the protein (Figure 4); hence, 190S and 192 T might participate in polysaccharide binding.

While we did not map the other sites responsible for just functional divergence and positive selection in this study, we analyzed the location of these sites based on the 3D structure of *GmEXPA1* (Table 8). The number of amino acid sites in domain II (the putative polysaccharide binding domain) responsible for either functional divergence or positive selection was clearly considerably greater than that in domain I, indicating the conservation of domain I compared to domain II. This difference might be associated with functional adaptiveness. Previous studies have shown that pollen EXPBs (group-1 allergens) have a marked loosening action on the cell walls of grasses, but not those of dicots; however, the reverse is true for EXPAs. Therefore, the two forms of expansin appear to target different components of the cell wall [13,92]. Consequently, the putative polysaccharide-binding domain (domain II) might have evolved to adapt to different components of the cell wall, thus promoting functional divergence and much faster evolution.

**Table 8:** Numbers of CAASs for functional divergence and positive selection in specific region of the protein structure

	**Type-I functional divergence**	**Type-II functional divergence**	**Site model of positive selection**	**Branch-site model of positive selection**	**Responsible for both functional divergence andpositive selection**
N terminus	3	6	2	1	1
Domain I	4	29	2	1	1
Linker	0	2	1	0	0
Domain II	11	26	9	9	5
C terminus	0	0	0	0	0

No sites responsible for functional divergence and positive selection were found in the C terminus, indicating that the C terminusis stringently conserved. In contrast, six amino acid sites responsible for functional divergence and three amino acid sites responsible for positive selection were found in the N terminus, indicating that this terminus contributes to functional divergence. In addition, the expansins of the N terminus are subject to variation, which might facilitate the adaptiveness of expansins for different functional needs. The N-terminal extension in EXPB1 from maize contained a motif (VPPG-PNITT) that was consistently found, with only minor variation, in group-1 grass pollen allergens, but not in other EXPBs [20]. While the function of this N-terminal extension is unknown, it may contribute to protein recognition, transport, packaging, and the processing of the pollen secretory apparatus [20].

## Conclusions

Previous studies have demonstrated that members of the expansin gene family play important roles in cell enlargement and a variety of other developmental processes. The results of the present study indicate that both tandem and segmental duplication have contributed to the expansion of the expansin gene family in soybean. Species-specific expansion characteristics were identified by comparing the expansion pattern of the expansin gene families in *Arabidopsis*, soybean, and rice. Segmental duplication seemed to be the predominant form of expansion for the expansin gene superfamilies of the two dicots, *Arabidopsis* and soybean. In contrast, tandem duplication seemed to be the predominant form of expansion for the expansin gene family of the monocot, rice. Furthermore, positive selection might be the main driving force for the functional divergence of duplicated genes, which might be critical for facilitating plant responses to various stressors throughout their evolutionary history. In addition, divergent selection constraints might have influenced the evolution of the four subfamilies. The results of this study are anticipated to further our understanding about the evolutionary processes of soybean expansin genes, and to help enhance functional genomic studies of expansins in an important model system.

## Methods

### Identification of expansin superfamily genes in soybean

Thirty-five gene sequences of the expansin superfamily in *Arabidopsis* were collected from EXPANSIN CENTRAL (http://www.personal.psu.edu/fsl/ExpCentral/), and used individually to blast against the soybean genome database in Phytozome v9.1 (http://www.phytozome.net/soybean). Sequences were selected as candidate proteins if their E value was ≤ 1e-10. Finally, the Pfam (http://www.sanger.ac.uk/Software/Pfam/) and the Simple Modular Architecture Research Tool (SMART; http://smart.embl-heidelberg.de/smart/batch.pl) were used to confirm each predicted expansin protein sequence was an expansin superfamily member, sharing domain I (PF03330) and domain II (PF01357). Redundant genes (genes with only one of the two domains, or with unintegrated ORF) were manually removed. Putative genes located on different chromosomes were found for each query sequence. A data file containing all the information from the target genes (including the locations on the chromosomes, genomic sequences, full CDS sequences, protein sequences, and 1500 bp of the nucleotide sequences upstream of the translation initiation codon) were downloaded from the website Phytozome (http://www.phytozome.net). The predicted possible signal peptides were estimated using the SignalP 4.1 server (http://www.cbs.dtu.dk/services/SignalP/). Theoretical pI (isoelectric point) and Mw (molecular weight) values were calculated by ExPASy Compute pI/Mw tool [93-95].

### Phylogenetic genetic tree construction and structural analysis

Construction of an unrooted neighbor-joining [96] phylogenetic tree and bootstrap analysis were conducted using the Molecular Evolutionary Genetics Analysis (MEGA) 5.0 program [97]. Motifs of paralogous expansin proteins were identified statistically using MEME with default settings; however, the maximum number of motifs to find was set at 10. Exon-intron organization of genes from the soybean expansin superfamily were determined by comparing predicted coding sequences (CDS) with their corresponding genomic sequences, using the online software GSDS (http://gsds.cbi.pku.edu.cn/).

### Analysis of expansin gene expansion patterns

Soybean expansin genes produced a scattered distribution pattern on chromosomes. In addition, several genes were clearly adjacent to one another based on their loci. Therefore, we focused on the process of segmental and tandem duplication. According to Schauser et al. [98], an effective way to detect a segmental duplication event is to identify additional paralogous protein pairs in the neighborhood of each family member. Consequently, the synteny blocks of each expansin member were searched in the Plant Genome Duplication Database [27]. Each expansin member was searched in the Plant Genome Duplication Database to identify whether it was involved in segmental duplication. Tandem duplications of the expansin genes in the soybean genome were identified by checking their physical locations on individual chromosomes. Tandem duplicated genes were defined as adjacent homologous genes on a single chromosome, with no more than one intervening gene. For example, *Glyma17g15670*/*Glyma17g15680*/*Glyma17g15690*/*Glyma17g15710* were identified as tandem duplicated gene clusters.

### Dating the duplication events

The Plant Genome Duplication Database directly provides the Ka and Ks with the corresponding duplicated gene pairs. When dating segmental duplication events, all available anchor points with Ks values between 0 and 1 were used to calculate the average Ks. However, duplicated gene pairs with fewer than three anchor points were deleted. The approximate date of the duplication event was calculated using the mean Ks values from T = Ks/2λ [99], in which the mean synonymous substitution rate (λ) for Fabaceae is 6.1 × 10^-9^[100]. For tandem duplication events, the protein sequences of the gene pairs were aligned in Clustal X 1.83, and PAL2NAL [101] was used to guide the resultant coding sequence (CDS) alignments. Ks, which is the number of synonymous substitutions per site, was determined using the aligned CDS in the Codeml procedure phylogenetic analysis by maximum likelihood (PAML) 4.4 [67] after all alignment gaps were eliminated. 4DTv, which is the transversion rate at four-fold synonymous codon positions, was also calculated by PAML at the same time.

### RNA-Seq atlas and promoter analysis

RNA-Seq data were introduced to further analyze the expression of expansin genes, and were obtained from Soybase (http://soybase.org/soyseq/) [102]. The *cis*-acting elements that regulate gene expression are distributed at 300–3000 bp upstream of the coding region, and sequence restriction was also taken into account in PlantCARE [36]. A total of 1500-bp nucleotide sequences upstream of the coding region for each soybean expansin gene were downloaded from Phytozome, and were submitted to PlantCARE for *insilico* analysis.

### Estimation of functional divergence

The software DIVERGE2 was used to detect the functional divergence between members of the soybean expansin subfamilies [103]. The coefficients of Type-I and Type-II functional divergence, θI and θII, between the soybean expansin subfamilies were calculated. If θI or θII is significantly greater than 0, it means that site-specific altered selective constraints or a radical shift of amino acid physiochemical property occurred after gene duplication and/or speciation [103]. Moreover, a site-specific posterior analysis was used to predict amino acid residues that were crucial for functional divergence. In this analysis, large posterior probability (Qk) indicates a high possibility that the functional constraint (or the evolutionary rate) and/or the radical change in the amino acid property of a site is different between two clusters [103].

### Tests of positive selection

Positive selection was investigated using a maximum likelihood approach by the Codeml procedure in PAML 4.4 [67], under the site model and branch site model. First, accurate nucleotide sequences and related multiple protein sequence alignments of the soybean expansins were obtained by PAL2NAL [101]. The resulting codon alignments and NJ tree were subsequently used in the Codeml program from the PAML package to calculate the dN/dS (or ω) ratio for each site, and to test different evolutionary models.

In the site model, two pairs of site models in PAML were chosen to test positive selection using the likelihood ratio test (LRT), and to identify positively selected sites in an orthologous group using both naive empirical Bayes (NEB) and Bayes empirical Bayes (BEB) estimation methods. First, models M0 (one ratio) and M3 (discrete) were compared, using a test for heterogeneity between codon sites in the dN/dS ratio value, ω. The second comparison was M7 (beta) vs M8 (beta + ω >1); this comparison is the most stringent test of positive selection [70]. When the LRT indicated positive selection, the BEB method was used to calculate the posterior probabilities that each codon is from the site class of positive selection under models M3 and M8 [72].

The branch site model assumes that the ω ratio varies between codon sites, and that there are four site classes in the sequence. The first class of sites is highly conserved in all lineages, with a small ω ratio, ω0. The second class includes neutral or weakly constrained sites, for which ω = ω1, where ω1 is near-to or smaller-than 1. In the third and fourth classes, the background lineages show ω0 or ω1, whereas the foreground branches show ω2, which may be greater than 1. When constructing the LRTs, the null hypothesis fixes ω2 = 1, allowing sites to evolve under the negative selection of the background lineages being released from constraint, and to evolve neutrally on the foreground lineage. The alternative hypothesis constrains ω2 ≥ 1 [72,104]. The posterior probabilities associated with specific codons falling into a site class affected by positive selection were calculated using the BEB method, described by Yang et al. [105].

### Availability of supporting data

The data sets supporting this article are included in:

Additional File 2. Protein sequences data of the expansin gene superfamilies in soybean, *Arabidopsis* and rice.

Additional File 3. Coding sequences data of the expansin gene superfamily in soybean.

Additional File 4. Genomic sequences data of the expansin gene superfamily in soybean.

Additional File 5. 1500 bp of the nucleotide sequences upstream of the translation initiation codon of the expanisn gene superfamily in soybean.

Additional File 8. The multiple sequence alignment of the soybean expansins.

Additional File 10. The RNA-Seq atlas data of the expansin genes.

## Competing interests

The authors declare that they have no competing interests.

## Authors’ contributions

YZ carried out the bioinformatic analysis and drafted the manuscript. YH designed the study and provide guidance on the whole study. NW, WS, and GY participated in the study and helped to draft the manuscript. YQ, YY coordinated the study and elaborated on manuscript. All authors read and approved the final manuscript.

## Supplementary Material

Additional file 1 The expansin gene superfamily in soybean. Click here for file

Additional file 2 **Protein sequences data of the expansin gene superfamilies in soybean, *Arabidopsis *** **and rice.** Click here for file

Additional file 3 Coding sequences data of the expansin gene superfamily in soybean. Click here for file

Additional file 4 Genomic sequences data of the expansin gene superfamily in soybean. Click here for file

Additional file 5 1500 bp of the nucleotide sequences data upstream of the translation initiation codon of the expanisn gene superfamily in soybean. Click here for file

Additional file 6 **Neighbor-joining phylogenetic tree of all of the expansin proteins in soybean, *Arabidopsis*, and rice.** Click here for file

Additional file 7 **The expansin gene superfamily in *Medicago truncatula *and *Phaseolus vulgaris*.** An neighbor-joining phylogenetic tree of all the expansin proteins in soybean, *Medicago truncatula* and *Phaseolus vulgaris* was provided. Clade of blue branches refers to the EXPA subfamily; clade of yellow branches refers to the EXPB subfamily; clade of green branches refers to the EXLA subfamily; clade of red braches refers to the EXLB subfamily. Genes from each subfamily of the expansin gene superfamily in *Medicago truncatula* and *Phaseolus vulgaris* were listed. Click here for file

Additional file 8 The multiple sequence alignment of the soybean expansins. Click here for file

Additional file 9 **Schematic diagram of the soybean expansin motifs.** The schematic diagram was derived from MEME. The ordering of the motifs of the expansin proteins in the diagram was automatically generated by MEME according to scores. Click here for file

Additional file 10 The RNA-Seq atlas data of the expansin genes. Click here for file

Additional file 11 **Expression pattern analysis based on the Libault Atlas.** The hierarchical cluster color code: the largest values are displayed as the reddest (hot), the smallest values are displayed as the bluest (cool), and the intermediate values are a lighter color of either blue or red. Pearson correlation clustering was used to group the developmentally regulated genes. Click here for file

Additional file 12 **Promoter analysis of the soybean expansin gene family.** The locus names,*cis*-acting element names, and mean number of different types of *cis*-element copies are listed. Click here for file

Additional file 13 **Expansion pattern of the expansin gene superfamily in *Arabidopsis*.** Click here for file

Additional file 14 Expansion pattern of the expansin gene superfamily in rice. Click here for file

## References

[B1] Cosgrove DJ Li LC Cho HT Hoffmann-Benning S Moore RC Blecker D The growing world of expansins Plant Cell Physiol 2002 43 12 1436 1444 10.1093/pcp/pcf180 12514240

[B2] McQueen-Mason S Durachko DM Cosgrove DJ Two endogenous proteins that induce cell wall extension in plants Plant Cell Online 1992 4 11 1425 1433 10.1105/tpc.4.11.1425 PMC160229 11538167

[B3] Li ZC Durachko DM Cosgrove DJ An oat coleoptile wall protein that induces wall extension in vitro and that is antigenically related to a similar protein from cucumber hypocotyls Planta 1993 191 3 349 356

[B4] Keller E Cosgrove DJ Expansins in growing tomato leaves Plant J 1995 8 6 795 802 10.1046/j.1365-313X.1995.8060795.x 11536718

[B5] Wu Y Sharp RE Durachko DM Cosgrove DJ Growth maintenance of the maize primary root at low water potentials involves increases in cell-wall extension properties, expansin activity, and wall susceptibility to expansins Plant Physiol 1996 111 3 765 772 11536740 10.1104/pp.111.3.765 PMC157893

[B6] Kende H Bradford K Brummell D Cho HT Cosgrove DJ Fleming AJ Gehring C Lee Y McQueen-Mason S Rose JKC Voesenek LACJ Nomenclature for members of the expansin superfamily of genes and proteins Plant Mol Biol 2004 55 3 311 314 10.1007/s11103-004-0158-6 15604683

[B7] Sampedro J Cosgrove DJ The expansin superfamily Genome Biol 2005 6 12 10.1186/gb-2005-6-12-242 PMC1414085 16356276

[B8] Sampedro J Carey RE Cosgrove DJ Genome histories clarify evolution of the expansin superfamily: new insights from the poplar genome and pine ESTs J Plant Res 2006 119 1 11 21 10.1007/s10265-005-0253-z 16411016

[B9] Cosgrove DJ Loosening of plant cell walls by expansins Nature 2000 407 6802 321 326 10.1038/35030000 11014181

[B10] Gray-Mitsumune M Mellerowicz EJ Abe H Schrader J Winzéll A Sterky F Blomqvist K McQueen-Mason S Teeri TT Sundberg B Expansins abundant in secondary xylem belong to subgroup A of the α-expansin gene family Plant Physiol 2004 135 3 1552 1564 10.1104/pp.104.039321 15247397 PMC519070

[B11] Belfield EJ Ruperti B Roberts JA McQueen-Mason S Changes in expansin activity and gene expression during ethylene-promoted leaflet abscission in *Sambucusnigra* J Exp Bot 2005 56 413 817 823 10.1093/jxb/eri076 15689341

[B12] Chen F Bradford KJ Expression of an expansin is associated with endosperm weakening during tomato seed germination Plant Physiol 2000 124 3 1265 1274 10.1104/pp.124.3.1265 11080302 PMC59224

[B13] Cosgrove DJ Bedinger P Durachko DM Group I allergens of grass pollen as cell wall-loosening agents Proc Natl Acad Sci 1997 94 12 6559 6564 10.1073/pnas.94.12.6559 9177257 PMC21089

[B14] Pezzotti M Feron R Mariani C Pollination modulates expression of the PPAL gene, a pistil-specific β-expansin Plant Mol Biol 2002 49 2 187 197 10.1023/A:1014962923278 11999374

[B15] Li LC Cosgrove DJ Grass group I pollen allergens (β-expansins) lack proteinase activity and do not cause wall loosening via proteolysis Eur J Biochem 2001 268 15 4217 4226 10.1046/j.1432-1327.2001.02336.x 11488915

[B16] McQueen-Mason SJ Cosgrove DJ Expansin mode of action on cell walls (analysis of wall hydrolysis, stress relaxation, and binding) Plant Physiol 1995 107 1 87 100 11536663 10.1104/pp.107.1.87 PMC161171

[B17] McQueen-Mason SJ Fry SC Durachko DM Cosgrove DJ The relationship between xyloglucanendotransglycosylase and in-vitro cell wall extension in cucumber hypocotyls Planta 1993 190 3 327 331 7763661 10.1007/BF00196961

[B18] Cosgrove DJ Relaxation in a high-stress environment: the molecular bases of extensible cell walls and cell enlargement Plant Cell 1997 9 7 1031 10.1105/tpc.9.7.1031 9254929 PMC156977

[B19] Kerff F Amoroso A Herman R Sauvagea E Petrellab S Filéea P Charliera P Jorisa B Tabuchic A Nikolaidisc N Cosgrovec DJ Crystal structure and activity of *Bacillus subtilis*YoaJ (EXLX1), a bacterial expansin that promotes root colonization Proc Natl Acad Sci 2008 105 44 16876 16881 10.1073/pnas.0809382105 18971341 PMC2579346

[B20] Yennawar NH Li LC Dudzinski DM Tabuchi A Cosgrove DJ Crystal structure and activities of EXPB1 (Zea m 1), a β-expansin and group-1 pollen allergen from maize Proc Natl Acad Sci 2006 103 40 14664 14671 10.1073/pnas.0605979103 16984999 PMC1595409

[B21] Schmutz J Cannon SB Schlueter J Ma J Mitros T Nelson W Hyten DL Song Q Thelen JJ Cheng J Xu D Hellsten U May GD Yu Y Sakurai T Umezawa T Bhattacharyya MK Sandhu D Valliyodan B Lindquist E Peto M Grant D Shu S Goodstein D Barry K Futrell-Griggs M Abernathy B Du J Tian Z Zhu L Genome sequence of the palaeopolyploid soybean Nature 2010 463 7278 178 183 10.1038/nature08670 20075913

[B22] Moore RC Purugganan MD The early stages of duplicate gene evolution Proc Natl Acad Sci 2003 100 26 15682 15687 10.1073/pnas.2535513100 14671323 PMC307628

[B23] Kong H Landherr LL Frohlich MW Leebens-Mack J Ma H DePamphilis CW Patterns of gene duplication in the plant *SKP1* gene family in angiosperms: evidence for multiple mechanisms of rapid gene birth Plant J 2007 50 5 873 885 10.1111/j.1365-313X.2007.03097.x 17470057

[B24] Cannon SB Mitra A Baumgarten A Young ND May G The roles of segmental and tandem gene duplication in the evolution of large gene families in *Arabidopsis thaliana* BMC Plant Biol 2004 4 1 10 10.1186/1471-2229-4-10 15171794 PMC446195

[B25] Yu J Wang J Lin W Li S Li H Zhou J Mi P Dong W Hu S Zeng C Zhang J Zhang Y Li R Xu Z Li S Li X Zheng H Cong L Lin L Yin J Geng J Li G Shi J Liu J Lv H Li J Wang J Deng Y Ran L Shi X The genomes of *Oryza sativa*: a history of duplications PLoS Biol 2005 3 2 e38 10.1371/journal.pbio.0030038 15685292 PMC546038

[B26] Ramamoorthy R Jiang SY Kumar N Venkatesh PN Ramachandran S A comprehensive transcriptional profiling of the WRKY gene family in rice under various abiotic and phytohormone treatments Plant Cell Physiol 2008 49 6 865 879 10.1093/pcp/pcn061 18413358

[B27] Tang H Wang X Bowers JE Ming R Alam M Paterson AH Unraveling ancient hexaploidy through multiply-aligned angiosperm gene maps Genome Res 2008 18 12 1944 1954 10.1101/gr.080978.108 18832442 PMC2593578

[B28] Du J Tian Z Sui Y Zhao M Song Q Cannon SB Cregan P Ma J Pericentromeric effects shape the patterns of divergence, retention, and expression of duplicated genes in the paleopolyploid soybean Plant Cell Online 2012 24 1 21 32 10.1105/tpc.111.092759 PMC3289580 22227891

[B29] Yin G Xu H Xiao S Qin Y Li Y Yan Y Hu Y The large soybean (*Glycine max*) WRKY TF family expanded by segmental duplication events and subsequent divergent selection among subgroups BMC Plant Biol 2013 13 1 148 10.1186/1471-2229-13-148 24088323 PMC3850935

[B30] Blanc G Wolfe KH Widespread paleopolyploidy in model plant species inferred from age distributions of duplicate genes Plant Cell Online 2004 16 7 1667 1678 10.1105/tpc.021345 PMC514152 15208399

[B31] Seoighe C Gehring C Genome duplication led to highly selective expansion of the*Arabidopsis thaliana*proteome Trends Genet 2004 20 10 461 464 10.1016/j.tig.2004.07.008 15363896

[B32] Maere S De Bodt S Raes J Casneuf T Van Montagu M Kuiper M Van de Peer Y Modeling gene and genome duplications in eukaryotes Proc Natl Acad Sci U S A 2005 102 15 5454 5459 10.1073/pnas.0501102102 15800040 PMC556253

[B33] Lee DK Ahn JH Song SK Do Choi Y Lee JS Expression of an expansin gene is correlated with root elongation in soybean Plant Physiol 2003 131 3 985 997 10.1104/pp.009902 12644651 PMC166864

[B34] Libault M Farmer A Joshi T Takahashi K Langley RJ Franklin LD He J Xu D May G Stacey G An integrated transcriptome atlas of the crop model Glycine max, and its use in comparative analyses in plants Plant J 2010 63 1 86 99 20408999 10.1111/j.1365-313X.2010.04222.x

[B35] Xue T Wang D Zhang S Ehlting J Ni F Jakab S Zheng C Zhong Y Genome-wide and expression analysis of protein phosphatase 2C in rice and Arabidopsis BMC Genomics 2008 9 1 550 10.1186/1471-2164-9-550 19021904 PMC2612031

[B36] Lescot M Déhais P Thijs G Marchal K Moreau Y Van de Peer Y Rouzé P Rombauts S PlantCARE, a database of plant *cis*-acting regulatory elements and a portal to tools for *in silico* analysis of promoter sequences Nucleic Acids Res 2002 30 1 325 327 10.1093/nar/30.1.325 11752327 PMC99092

[B37] Sommer H Saedler H Structure of the chalcone synthase gene of *Antirrhinum majus* Mol Gen Genet MGG 1986 202 3 429 434 10.1007/BF00333273

[B38] Menkens AE Schindler U Cashmore AR The G-box: a ubiquitous regulatory DNA element in plants bound by the GBF family of bZIP proteins Trends Biochem Sci 1995 20 12 506 510 10.1016/S0968-0004(00)89118-5 8571452

[B39] Lois R Dietrich A Hahlbrock K Schulz W A phenylalanine ammonia-lyase gene from parsley: structure, regulation and identification of elicitor and light responsive *cis*-acting elements EMBO J 1989 8 6 1641 2767049 10.1002/j.1460-2075.1989.tb03554.x PMC401003

[B40] Arguello-Astorga GR Herrera-Estrella LR Ancestral multipartite units in light-responsive plant promoters have structural features correlating with specific phototransduction pathways Plant Physiol 1996 112 3 1151 1166 10.1104/pp.112.3.1151 8938415 PMC158042

[B41] Pastuglia M Roby D Dumas C Cock JM Rapid induction by wounding and bacterial infection of an S gene family receptor-like kinase gene in Brassica oleracea Plant Cell Online 1997 9 1 49 60 10.1105/tpc.9.1.49 PMC156900 9014364

[B42] Guilfoyle TJ Hagen G Li Y Ulmasov T Liu ZB Strabala T Gee M Auxin-regulated transcription Funct Plant Biol 1993 20 5 489 502

[B43] Ogawa M Hanada A Yamauchi Y Kuwahara A Kamiya Y Yamaguchi S Gibberellin biosynthesis and response during Arabidopsis seed germination Plant Cell Online 2003 15 7 1591 1604 10.1105/tpc.011650 PMC165403 12837949

[B44] Ballas N Wong LM Ke M Theologis A Two auxin-responsive domains interact positively to induce expression of the early indoleacetic acid-inducible gene PS-IAA4/5 Proc Natl Acad Sci 1995 92 8 3483 3487 10.1073/pnas.92.8.3483 7724586 PMC42191

[B45] Pascuzzi P Hamilton D Bodily K Arias J Auxin-induced stress potentiates *trans*-activation by a conserved plant basic/leucine-zipper factor J Biol Chem 1998 273 41 26631 26637 10.1074/jbc.273.41.26631 9756903

[B46] Kim JK Cao J Wu R Regulation and interaction of multiple protein factors with the proximal promoter regions of a rice high pl α-amylase gene Mol Gen Genet MGG 1992 232 3 383 393 10.1007/BF00266241 1375314

[B47] Jacobsen JV Gu B Davies PJ Pp 246-271bler F, Chandler PM. **Gibberellin and abscisic acid in germinating cereals** Plant hormones: physiology, biochemistry and molecular biology 1995 Dordrecht, The Netherlands: Kluwer Academic

[B48] Catalá C Rose JKC Bennett AB Auxin-regulated genes encoding cell wall-modifying proteins are expressed during early tomato fruit growth Plant Physiol 2000 122 2 527 534 10.1104/pp.122.2.527 10677445 PMC58889

[B49] Hutchison KW Singer PB McInnis S Diaz-Sala C Greenwood MS Expansins are conserved in conifers and expressed in hypocotyls in response to exogenous auxin Plant Physiol 1999 120 3 827 832 10.1104/pp.120.3.827 10398718 PMC59321

[B50] Cho HT Kende H Expression of expansin genes is correlated with growth in deepwater rice Plant Cell Online 1997 9 9 1661 1671 10.1105/tpc.9.9.1661 PMC157041 9338967

[B51] Lee Y Kende H Expression of β-expansins is correlated with internodal elongation in deepwater rice Plant Physiol 2001 127 2 645 654 10.1104/pp.010345 11598238 PMC125099

[B52] Klotz KL Lagrimini LM Phytohormone control of the tobacco anionic peroxidase promoter Plant Mol Biol 1996 31 3 565 573 10.1007/BF00042229 8790289

[B53] Yamaguchi-Shinozaki K Shinozaki K *Arabidopsis* DNA encoding two desiccation-responsive *rd*29 genes Plant Physiol 1993 101 3 1119 10.1104/pp.101.3.1119 8310052 PMC158736

[B54] Freitas FZ Bertolini MC Genomic organization of the *Neurosporacrassagsn* gene: possible involvement of the STRE and HSE elements in the modulation of transcription during heat shock Mol Genet Genomics 2004 272 5 550 561 10.1007/s00438-004-1086-5 15558319

[B55] Wu Y Meeley RB Cosgrove DJ Analysis and expression of the α-expansin and β-expansin gene families in maize Plant Physiol 2001 126 1 222 232 10.1104/pp.126.1.222 11351085 PMC102296

[B56] Jones L McQueen-Mason S A role for expansins in dehydration and rehydration of the resurrection plant *Craterostigmaplantagineum* FEBS Lett 2004 559 1 61 65 14960308 10.1016/S0014-5793(04)00023-7

[B57] Cho HT Kende H Expansins in deepwater rice internodes Plant Physiol 1997 113 4 1137 1143 10.1104/pp.113.4.1137 9112771 PMC158236

[B58] Huang J Takano T Akita S Expression of α-expansin genes in young seedlings of rice (*Oryza sativa* L.) Planta 2000 211 4 467 473 10.1007/s004250000311 11030545

[B59] Kim JH Cho HT Kende H α-Expansins in the semiaquatic ferns *Marsileaquadrifolia* and *Regnellidiumdiphyllum*: evolutionary aspects and physiological role in rachis elongation Planta 2000 212 1 85 92 10.1007/s004250000367 11219587

[B60] Vriezen WH De Graaf B Mariani C Voesenek LA Submergence induces expansin gene expression in flooding-tolerant *Rumexpalustris* and not in flooding-intolerant *R. acetosa* Planta 2000 210 6 956 963 10.1007/s004250050703 10872228

[B61] Colmer TD Peeters AJM Wagemaker CAM Vriezen WH Ammerlaan A Voesenek LACJ Expression of α-expansin genes during root acclimations to O2 deficiency in *Rumexpalustris* Plant Mol Biol 2004 56 3 423 437 10.1007/s11103-004-3844-5 15604754

[B62] Pichersky E Bernatzky R Tanksley SD Breidenbach RB Kausch AP Cashmore AR Molecular characterization and genetic mapping of two clusters of genes encoding chlorophyll*a/b*-binding proteins in*Lycopersiconesculentum* (tomato) Gene 1985 40 2 247 258 3007291 10.1016/0378-1119(85)90047-2

[B63] Yamaji N Ma JF Spatial distribution and temporal variation of the rice silicon transporter Lsi1 Plant Physiol 2007 143 3 1306 1313 10.1104/pp.106.093005 17259286 PMC1820904

[B64] Liu Q Wang H Zhang Z Wu J Feng Y Zhu Z Divergence in function and expression of the NOD26-like intrinsic proteins in plants BMC Genomics 2009 10 1 313 10.1186/1471-2164-10-313 19604350 PMC2726226

[B65] Liu Q Zhu H Molecular evolution of the*MLO*gene family in *Oryza sativa*and their functional divergence Gene 2008 409 1 1 10 18155857 10.1016/j.gene.2007.10.031

[B66] Wang M Wang Q Zhao H Zhang X Pan Y Evolutionary selection pressure of forkhead domain and functional divergence Gene 2009 432 1 19 25 19100316 10.1016/j.gene.2008.11.018

[B67] Yang Z PAML 4: phylogenetic analysis by maximum likelihood Mol Biol Evol 2007 24 8 1586 1591 10.1093/molbev/msm088 17483113

[B68] Yang Z Nielsen R Estimating synonymous and nonsynonymous substitution rates under realistic evolutionary models Mol Biol Evol 2000 17 1 32 43 10.1093/oxfordjournals.molbev.a026236 10666704

[B69] Yang Z PAML: Phylogenetic analysis by maximum likelihood Version 3.14 2004 London: University College London

[B70] Anisimova M Bielawski JP Yang Z Accuracy and power of the likelihood ratio test in detecting adaptive molecular evolution Mol Biol Evol 2001 18 8 1585 1592 10.1093/oxfordjournals.molbev.a003945 11470850

[B71] Yang Z PAML: a program package for phylogenetic analysis by maximum likelihood Comput Appl Biosci 1997 13 5 555 556 9367129 10.1093/bioinformatics/13.5.555

[B72] Zhang J Nielsen R Yang Z Evaluation of an improved branch-site likelihood method for detecting positive selection at the molecular level Mol Biol Evol 2005 22 12 2472 2479 10.1093/molbev/msi237 16107592

[B73] Otto SP Whitton J Polyploid incidence and evolution Annu Rev Genet 2000 34 1 401 437 10.1146/annurev.genet.34.1.401 11092833

[B74] Blanc G Hokamp K Wolfe KH A recent polyploidy superimposed on older large-scale duplications in the *Arabidopsis* genome Genome Res 2003 13 2 137 144 10.1101/gr.751803 12566392 PMC420368

[B75] Bowers JE Chapman BA Rong J Paterson AH Unravelling angiosperm genome evolution by phylogenetic analysis of chromosomal duplication events Nature 2003 422 6930 433 438 10.1038/nature01521 12660784

[B76] Paterson AH Bowers JE Chapman BA Ancient polyploidization predating divergence of the cereals, and its consequences for comparative genomics Proc Natl Acad Sci U S A 2004 101 26 9903 9908 10.1073/pnas.0307901101 15161969 PMC470771

[B77] Force A Lynch M Pickett FB Amores A Yan YL Postlethwait J Preservation of duplicate genes by complementary, degenerative mutations Genetics 1999 151 4 1531 1545 10101175 10.1093/genetics/151.4.1531 PMC1460548

[B78] Lynch M Force A The probability of duplicate gene preservation bysubfunctionalization Genetics 2000 154 1 459 473 10629003 10.1093/genetics/154.1.459 PMC1460895

[B79] He X Zhang J Gene complexity and gene duplicability Curr Biol 2005 15 11 1016 1021 10.1016/j.cub.2005.04.035 15936271

[B80] Sémon M Wolfe KH Consequences of genome duplication Curr Opin Genet Dev 2007 17 6 505 512 10.1016/j.gde.2007.09.007 18006297

[B81] Schnable JC Springer NM Freeling M Differentiation of the maize subgenomes by genome dominance and both ancient and ongoing gene loss Proc Natl Acad Sci 2011 108 10 4069 4074 10.1073/pnas.1101368108 21368132 PMC3053962

[B82] Freeling M The evolutionary position of subfunctionalization, downgraded 2008 10.1159/000126004 18756075

[B83] Hanada K Zou C Lehti-Shiu MD Shinozaki K Shiu SH Importance of lineage-specific expansion of plant tandem duplicates in the adaptive response to environmental stimuli Plant Physiol 2008 148 2 993 1003 10.1104/pp.108.122457 18715958 PMC2556807

[B84] Cosgrove DJ New genes and new biological roles for expansins Curr Opin Plant Biol 2000 3 1 73 79 10.1016/S1369-5266(99)00039-4 10679451

[B85] Zheng Y Xu D Gu X Functional divergence after gene duplication and sequence–structure relationship: a case study of G‒protein alpha subunits J Exp Zool B Mol Dev Evol 2007 308 1 85 96 17094082 10.1002/jez.b.21140

[B86] Gu X Zhang Z Huang W Rapid evolution of expression and regulatory divergences after yeast gene duplication Proc Natl Acad Sci U S A 2005 102 3 707 712 10.1073/pnas.0409186102 15647348 PMC545572

[B87] Ha M Kim ED Chen ZJ Duplicate genes increase expression diversity in closely related species and allopolyploids Proc Natl Acad Sci 2009 106 7 2295 2300 10.1073/pnas.0807350106 19168631 PMC2650150

[B88] Fetterman CD Rannala B Walter MA Identification and analysis of evolutionary selection pressures acting at the molecular level in five forkhead subfamilies BMC Evol Biol 2008 8 1 261 18816404 10.1186/1471-2148-8-261 PMC2570691

[B89] Arnold K Bordoli L Kopp J Schwede T The SWISS-MODEL workspace: a web-based environment for protein structure homology modelling Bioinformatics 2006 22 2 195 201 10.1093/bioinformatics/bti770 16301204

[B90] Kiefer F Arnold K Künzli M Bordoli L Schwede T The SWISS-MODEL Repository and associated resources Nucleic Acids Res 2009 37 suppl 1 D387 D392 18931379 10.1093/nar/gkn750 PMC2686475

[B91] Peitsch M Protein modeling by E-mail Biotechnology 1995 13 658 660 10.1038/nbt0795-658

[B92] Li LC Bedinger PA Volk C Jones AD Cosgrove DJ Purification and characterization of four β-expansins (Zea m 1 isoforms) from maize pollen Plant Physiol 2003 132 4 2073 2085 10.1104/pp.103.020024 12913162 PMC181291

[B93] Bjellqvist B Hughes GJ Pasquali C Paquet N Ravier F Sanchez JC Frutiger S Hochstrasser D The focusing positions of polypeptides in immobilized pH gradients can be predicted from their amino acid sequences Electrophoresis 1993 14 1 1023 1031 10.1002/elps.11501401163 8125050

[B94] Bjellqvist B Basse B Olsen E Celis JE Reference points for comparisons of two‒dimensional maps of proteins from different human cell types defined in a pH scale where isoelectric points correlate with polypeptide compositions Electrophoresis 1994 15 1 529 539 10.1002/elps.1150150171 8055880

[B95] Gasteiger E Hoogland C Gattiker A Wikins MR Appel RD Bairoch A Protein identification and analysis tools on the ExPASy server[M]//The proteomics protocols handbook 2005 USA: Humana Press

[B96] Saitou N Nei M The neighbor-joining method: a new method for reconstructing phylogenetic trees Mol Biol Evol 1987 4 4 406 425 3447015 10.1093/oxfordjournals.molbev.a040454

[B97] Tamura K Peterson D Peterson N Stecher G Nei M Kumar S MEGA5: molecular evolutionary genetics analysis using maximum likelihood, evolutionary distance, and maximum parsimony methods Mol Biol Evol 2011 28 10 2731 2739 10.1093/molbev/msr121 21546353 PMC3203626

[B98] Schauser L Wieloch W Stougaard J Evolution of NIN-like proteins in *Arabidopsis*, rice, and *Lotus japonicus* J Mol Evol 2005 60 2 229 237 10.1007/s00239-004-0144-2 15785851

[B99] Nei M Kumar S Molecular evolution and phylogenetics 2000 Oxford: Oxford University Press

[B100] Lynch M Conery JS The evolutionary fate and consequences of duplicate genes Science 2000 290 5494 1151 1155 10.1126/science.290.5494.1151 11073452

[B101] Suyama M Torrents D Bork P PAL2NAL: robust conversion of protein sequence alignments into the corresponding codon alignments Nucleic Acids Res 2006 34 suppl 2 W609 W612 16845082 10.1093/nar/gkl315 PMC1538804

[B102] Severin AJ Woody JL Bolon YT Joseph B Diers BW Farmer AD Muehlbauer GJ Nelson RT Grant D Specht JE Graham MA Cannon SB May GD Vance CP Shoemaker RC RNA-Seq Atlas of *Glycine max*: a guide to the soybean transcriptome BMC Plant Biol 2010 10 1 160 10.1186/1471-2229-10-160 20687943 PMC3017786

[B103] Gu X A simple statistical method for estimating type-II (cluster-specific) functional divergence ofprotein sequences Mol Biol Evol 2006 23 10 1937 1945 10.1093/molbev/msl056 16864604

[B104] Yang Z Nielsen R Codon-substitution models for detecting molecular adaptation at individual sites along specific lineages Mol Biol Evol 2002 19 6 908 917 10.1093/oxfordjournals.molbev.a004148 12032247

[B105] Yang Z Wong WSW Nielsen R Bayes empirical Bayes inference of amino acid sites under positive selection Mol Biol Evol 2005 22 4 1107 1118 10.1093/molbev/msi097 15689528

