# Supplementary material for: Soybean (Glycine max) expansin gene superfamily origins: segmental and tandem duplication events followed by divergent selection among subfamilies
Source: BMC Plant Biol. 2014 Apr 11;14:93. doi: 10.1186/1471-2229-14-93 (PMC4021193; doi:10.1186/1471-2229-14-93)
Supplement: Additional file 1 — The expansin gene superfamily in soybean. [file 1471-2229-14-93-S1.docx]

**Additional File 1**: The expansin gene superfamily

| Gene name | Gene locus | Chromosome | Protein length(aa) | Intron No. | Signal peptide(aa) | pI | Mw(kD) |
| --- | --- | --- | --- | --- | --- | --- | --- |
| *GmEXPA1* | Glyma01g06030 | 1 | 250 | 2 | 20 | 9.3 | 26.9 |
| *GmEXPA2* | Glyma01g42370 | 1 | 260 | 2 | 26 | 9.4 | 28.6 |
| *GmEXPA3* | Glyma02g12140 | 2 | 250 | 3 | 20 | 9.3 | 26.9 |
| *GmEXPA4* | Glyma02g40230 | 2 | 254 | 2 | 27 | 8.4 | 27.2 |
| *GmEXPA5* | Glyma02g40790 | 2 | 270 | 1 | 16 | 8.1 | 29.2 |
| *GmEXPA6* | Glyma02g41590 | 2 | 257 | 1 | 20 | 9.5 | 27.6 |
| *GmEXPA7* | Glyma03g04390 | 3 | 249 | 2 | 25 | 9.6 | 26.8 |
| *GmEXPA8* | Glyma03g38480 | 3 | 255 | 2 | 26 | 9.6 | 27.8 |
| *GmEXPA9* | Glyma04g02380 | 4 | 256 | 2 | 24 | 8.6 | 27.6 |
| *GmEXPA10* | Glyma04g33350 | 4 | 248 | 2 | 20 | 9.0 | 26.7 |
| *GmEXPA11* | Glyma04g40000 | 4 | 250 | 2 | 22 | 8.4 | 26.8 |
| *GmEXPA12* | Glyma05g03720 | 5 | 229 | 3 | None | 8.8 | 24.8 |
| *GmEXPA13* | Glyma06g02420 | 6 | 255 | 2 | 23 | 8.8 | 27.4 |
| *GmEXPA14* | Glyma06g02430 | 6 | 252 | 2 | 18 | 9.1 | 27.3 |
| *GmEXPA15* | Glyma06g14850 | 6 | 250 | 2 | 22 | 8.4 | 26.8 |
| *GmEXPA16* | Glyma06g20970 | 6 | 249 | 2 | 18 | 9.1 | 26.7 |
| *GmEXPA17* | Glyma06g38100 | 6 | 257 | 2 | 19 | 9.6 | 28.5 |
| *GmEXPA18* | Glyma06g44940 | 6 | 254 | 2 | 21 | 8.0 | 28.3 |
| *GmEXPA19* | Glyma07g15910 | 7 | 258 | 2 | 21 | 9.8 | 27.8 |
| *GmEXPA20* | Glyma07g35620 | 7 | 248 | 3 | 20 | 9.3 | 26.5 |
| *GmEXPA21* | Glyma09g37090 | 9 | 265 | 3 | None | 9.5 | 29.1 |
| *GmEXPA22* | Glyma10g28040 | 10 | 254 | 2 | None | 9.4 | 27.7 |
| *GmEXPA23* | Glyma11g03000 | 11 | 260 | 2 | 25 | 9.6 | 28.5 |
| *GmEXPA24* | Glyma11g14800 | 11 | 259 | 2 | 22 | 9.6 | 28.1 |
| *GmEXPA25* | Glyma11g26240 | 11 | 255 | 2 | 28 | 7.6 | 27.4 |
| *GmEXPA26* | Glyma11g34040 | 11 | 258 | 2 | 21 | 9.6 | 28.1 |
| *GmEXPA27* | Glyma12g06730 | 12 | 259 | 2 | 22 | 9.6 | 28.2 |
| *GmEXPA28* | Glyma12g12340 | 12 | 254 | 2 | 16 | 8.3 | 28.3 |
| *GmEXPA29* | Glyma12g23200 | 12 | 256 | 2 | 28 | 9.7 | 28.6 |
| *GmEXPA30* | Glyma13g41160 | 13 | 257 | 1 | 20 | 9.5 | 27.9 |
| *GmEXPA31* | Glyma14g07360 | 14 | 260 | 1 | 23 | 9.5 | 28.0 |
| *GmEXPA32* | Glyma14g38430 | 14 | 254 | 2 | 27 | 7.6 | 27.3 |
| *GmEXPA33* | Glyma14g39120 | 14 | 266 | 1 | 25 | 8.6 | 28.9 |
| *GmEXPA34* | Glyma15g04240 | 15 | 256 | 2 | 19 | 9.4 | 27.8 |
| *GmEXPA35* | Glyma17g10950 | 17 | 267 | 3 | None | 8.4 | 28.8 |
| *GmEXPA36* | Glyma17g14230 | 17 | 309 | 2 | None | 8.8 | 33.8 |
| *GmEXPA37* | Glyma17g37990 | 17 | 255 | 2 | 23 | 9.1 | 27.5 |
| *GmEXPA38* | Glyma18g04260 | 18 | 262 | 2 | 25 | 9.7 | 28.6 |
| *GmEXPA39* | Glyma18g05040 | 18 | 281 | 1 | 23 | 5.5 | 30.2 |
| *GmEXPA40* | Glyma18g06063 | 18 | 255 | 2 | 27 | 7.6 | 27.3 |
| *GmEXPA41* | Glyma18g25160 | 18 | 258 | 2 | 21 | 9.7 | 27.6 |
| *GmEXPA42* | Glyma18g39850 | 18 | 258 | 2 | 21 | 9.7 | 27.8 |
| *GmEXPA43* | Glyma18g49570 | 18 | 265 | 3 | None | 9.5 | 29.1 |
| *GmEXPA44* | Glyma18g50030 | 18 | 228 | 2 | 22 | 8.7 | 25.2 |
| *GmEXPA45* | Glyma19g02810 | 19 | 259 | 2 | None | 9.3 | 28.5 |
| *GmEXPA46* | Glyma19g37060 | 19 | 287 | 2 | 28 | 9.3 | 31.6 |
| *GmEXPA47* | Glyma19g41080 | 19 | 254 | 2 | 24 | 9.7 | 27.9 |
| *GmEXPA48* | Glyma20g04490 | 20 | 265 | 2 | None | 9.4 | 28.7 |
| *GmEXPA49* | Glyma20g22050 | 20 | 254 | 2 | 25 | 9.4 | 27.8 |
| *GmEXPB1* | Glyma01g16140 | 1 | 277 | 3 | None | 4.9 | 29.5 |
| *GmEXPB2* | Glyma03g03980 | 3 | 267 | 3 | 24 | 5.6 | 28.8 |
| *GmEXPB3* | Glyma06g44930 | 6 | 267 | 3 | 31 | 8.7 | 29.2 |
| *GmEXPB4* | Glyma10g24080 | 10 | 277 | 3 | 29 | 5.4 | 29.5 |
| *GmEXPB5* | Glyma10g24120 | 10 | 272 | 3 | 29 | 7.5 | 29.1 |
| *GmEXPB6* | Glyma11g17160 | 11 | 277 | 3 | None | 5.0 | 29.4 |
| *GmEXPB7* | Glyma12g12350 | 12 | 267 | 3 | 31 | 8.7 | 29.1 |
| *GmEXPB8* | Glyma12g33070 | 12 | 261 | 3 | 23 | 8.3 | 28.3 |
| *GmEXPB9* | Glyma13g37395 | 13 | 258 | 3 | 23 | 9.1 | 28.2 |
| *GmEXLA1* | Glyma11g10240 | 11 | 259 | 4 | 19 | 8.3 | 28.0 |
| *GmEXLA2* | Glyma12g02550 | 12 | 261 | 4 | 19 | 8.5 | 28.3 |
| *GmEXLB1* | Glyma01g35070 | 1 | 218 | 5 | 20 | 4.6 | 23.5 |
| *GmEXLB2* | Glyma01g41050 | 1 | 255 | 4 | 20 | 4.5 | 27.8 |
| *GmEXLB3* | Glyma01g41330 | 1 | 251 | 3 | 24 | 5.3 | 28.2 |
| *GmEXLB4* | Glyma05g05390 | 5 | 244 | 3 | 24 | 9.0 | 27.0 |
| *GmEXLB5* | Glyma05g05420 | 5 | 247 | 3 | 24 | 8.3 | 27.5 |
| *GmEXLB6* | Glyma05g05430 | 5 | 251 | 3 | 24 | 5.6 | 27.9 |
| *GmEXLB7* | Glyma05g05880 | 5 | 250 | 4 | 20 | 5.1 | 27.0 |
| *GmEXLB8* | Glyma11g04080 | 11 | 251 | 3 | 24 | 5.5 | 28.2 |
| *GmEXLB9* | Glyma11g04370 | 11 | 255 | 4 | 21 | 4.7 | 28.0 |
| *GmEXLB10* | Glyma17g15640 | 17 | 250 | 4 | 24 | 9.4 | 27.5 |
| *GmEXLB11* | Glyma17g15670 | 17 | 250 | 3 | 24 | 9.4 | 27.5 |
| *GmEXLB12* | Glyma17g15680 | 17 | 248 | 3 | 24 | 9.2 | 27.4 |
| *GmEXLB13* | Glyma17g15690 | 17 | 247 | 3 | 24 | 6.3 | 27.7 |
| *GmEXLB14* | Glyma17g15710 | 17 | 251 | 3 | 24 | 5.4 | 27.9 |
| *GmEXLB15* | Glyma17g16210 | 17 | 251 | 4 | 21 | 4.9 | 27.2 |
