# Supplementary figures and images for: Soybean (Glycine max) expansin gene superfamily origins: segmental and tandem duplication events followed by divergent selection among subfamilies
Source: BMC Plant Biol. 2014 Apr 11;14:93. doi: 10.1186/1471-2229-14-93 (PMC4021193; doi:10.1186/1471-2229-14-93)

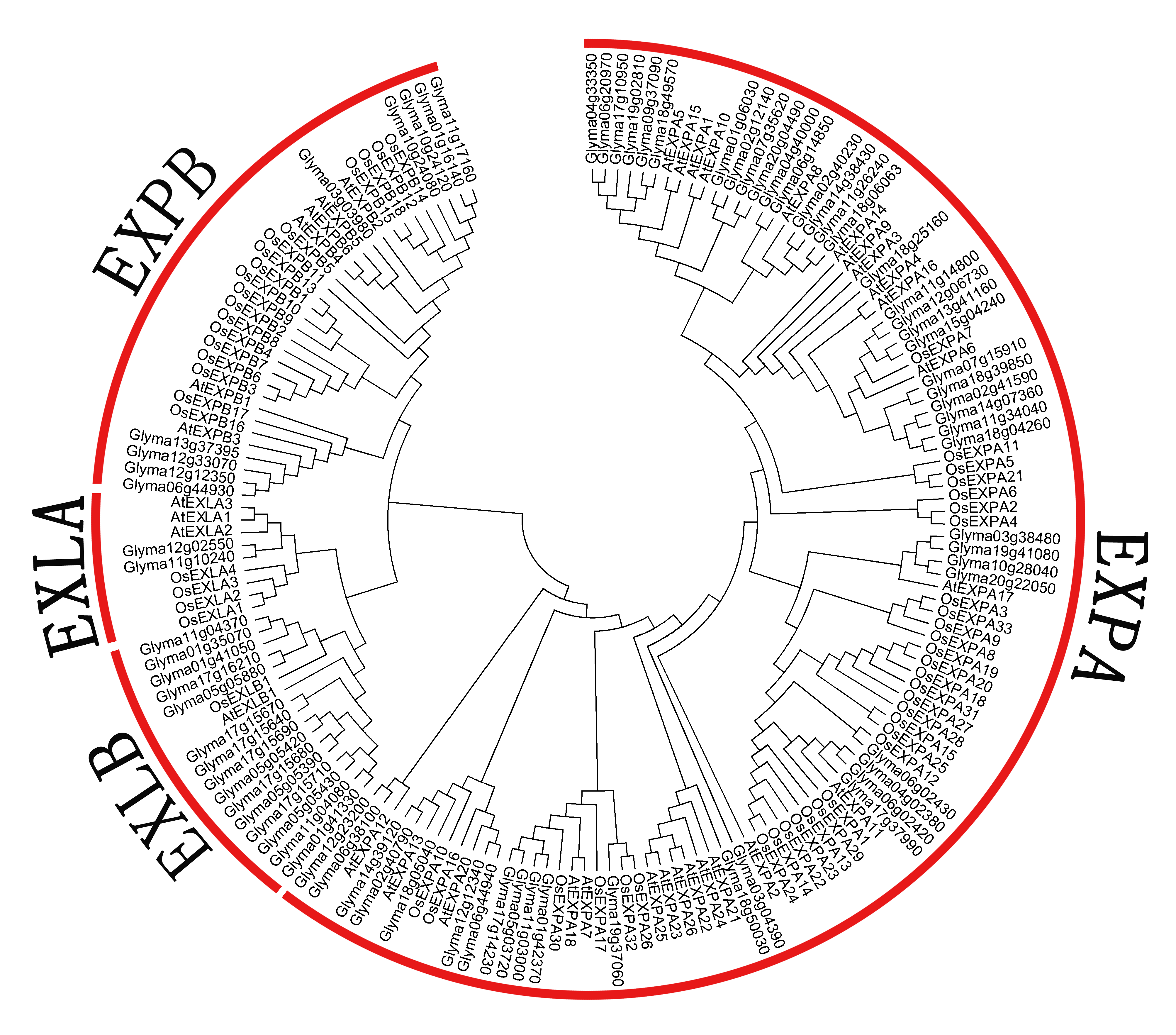

Supplement: Additional file 6 — Neighbor-joining phylogenetic tree of all of the expansin proteins in soybean, Arabidopsis , and rice. [file 1471-2229-14-93-S6.png]

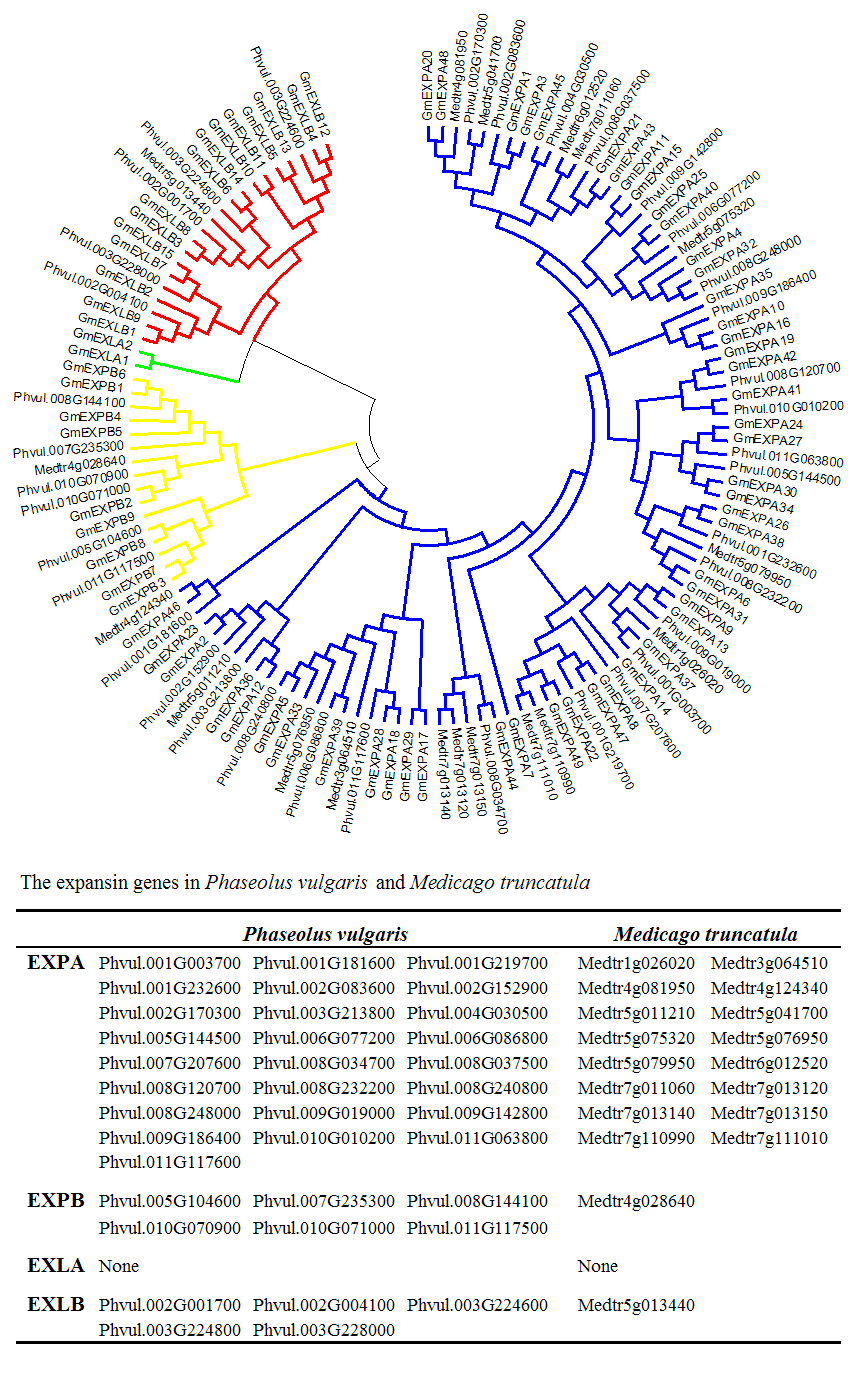

Supplement: Additional file 7 — The expansin gene superfamily in Medicago truncatula and Phaseolus vulgaris. An neighbor-joining phylogenetic tree of all the expansin proteins in soybean, Medicago truncatula and Phaseolus vulgaris was provided. Clade of blue branches refers to the EXPA subfamily; clade of yellow branches refers to the EXPB subfamily; clade of green branches refers to the EXLA subfamily; clade of red braches refers to the EXLB subfamily. Genes from each subfamily of the expansin gene superfamily in Medicago truncatula and Phaseolus vulgaris were listed. [file 1471-2229-14-93-S7.tiff]

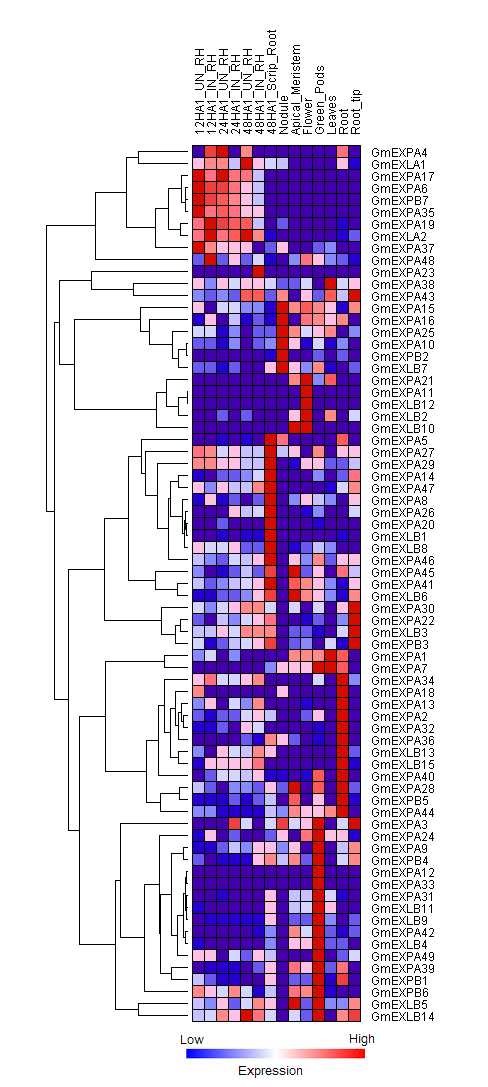

Supplement: Additional file 11 — Expression pattern analysis based on the Libault Atlas. The hierarchical cluster color code: the largest values are displayed as the reddest (hot), the smallest values are displayed as the bluest (cool), and the intermediate values are a lighter color of either blue or red. Pearson correlation clustering was used to group the developmentally regulated genes. [file 1471-2229-14-93-S11.tiff]
