# Supplementary material for: Soybean (Glycine max) expansin gene superfamily origins: segmental and tandem duplication events followed by divergent selection among subfamilies
Source: BMC Plant Biol. 2014 Apr 11;14:93. doi: 10.1186/1471-2229-14-93 (PMC4021193; doi:10.1186/1471-2229-14-93)

**Additional File 9:** Schematic diagram of motifs of soybean expansin proteins.

The schematic diagram was derived from MEME. The order of motifs of expansin proteins in the diagram was automatically generated by MEME according to scores.


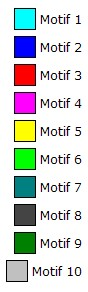


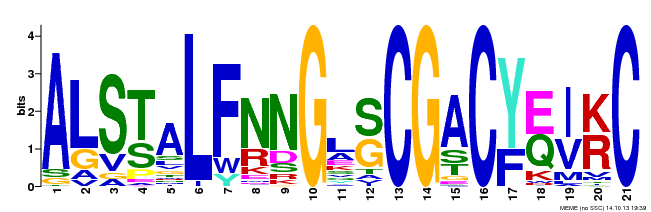


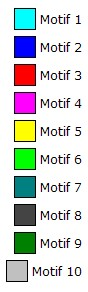


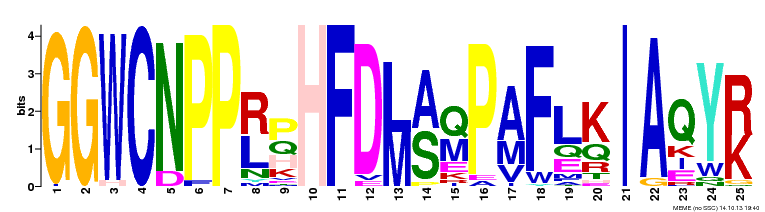


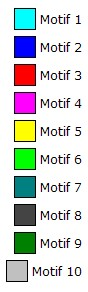


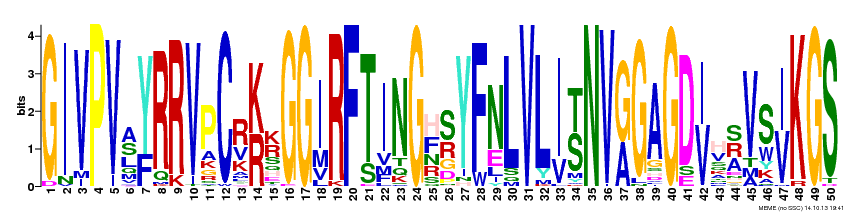


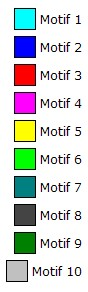


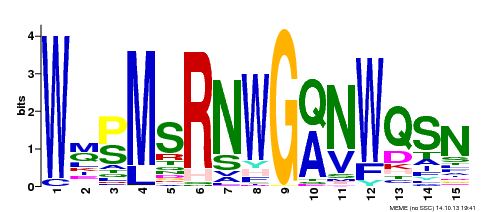


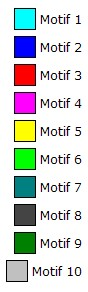


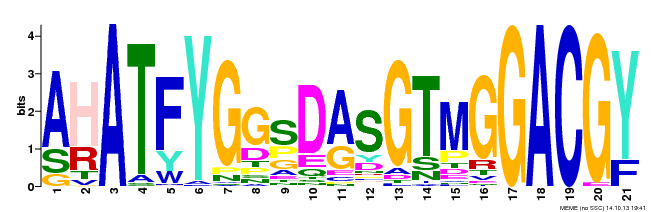


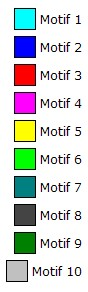


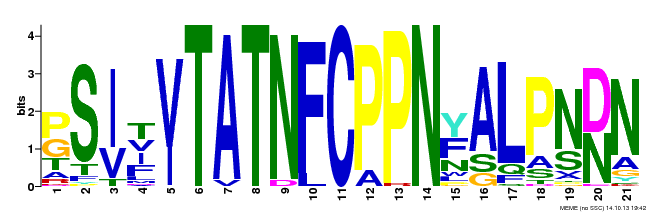


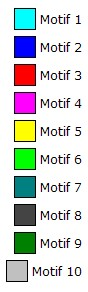


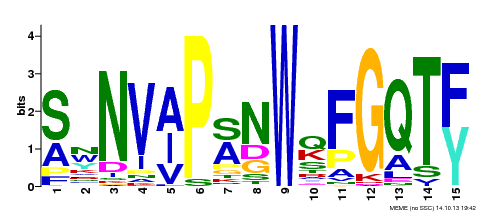


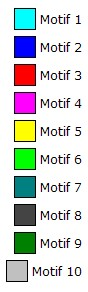


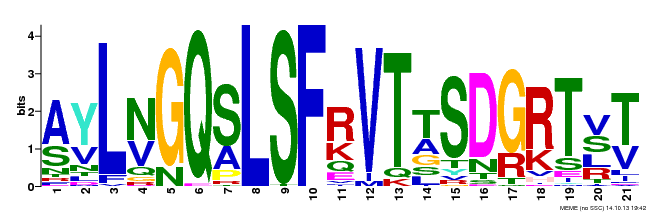


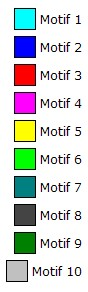


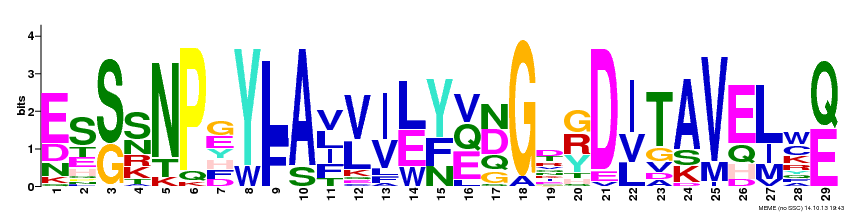


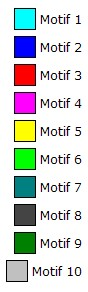


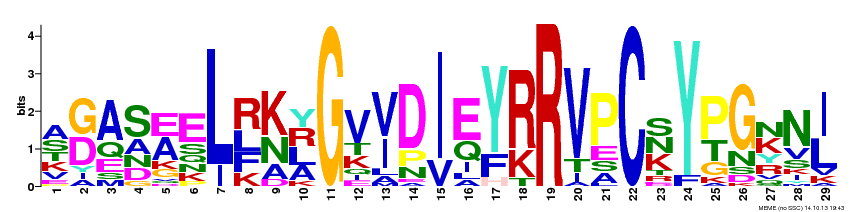

Supplement: Additional file 9 — Schematic diagram of the soybean expansin motifs. The schematic diagram was derived from MEME. The ordering of the motifs of the expansin proteins in the diagram was automatically generated by MEME according to scores. [file 1471-2229-14-93-S9.docx]
