# Supplementary material for: Soybean (Glycine max) expansin gene superfamily origins: segmental and tandem duplication events followed by divergent selection among subfamilies
Source: BMC Plant Biol. 2014 Apr 11;14:93. doi: 10.1186/1471-2229-14-93 (PMC4021193; doi:10.1186/1471-2229-14-93)
Supplement: Additional file 13 — Expansion pattern of the expansin gene superfamily in Arabidopsis . [file 1471-2229-14-93-S13.docx]

Additional File 13: Expansion pattern of the expansin gene superfamily in *Arabidopsis*.

**Segemental duplicated gene pairs:**

| Segemental duplicated gene pairs | Subfamily |
| --- | --- |
| AT1G12560 & AT1G62980 | EXPA |
| AT1G26770 & AT3G29030 | EXPA |
| AT1G26770 & AT2G03090 | EXPA |
| AT1G26770 & AT1G69530 | EXPA |
| AT1G69530 & AT3G29030 | EXPA |
| AT1G69530 & AT2G03090 | EXPA |
| AT3G55500 & AT5G02260 | EXPA |
| AT2G28950 & AT3G55500 | EXPA |
| AT2G28950 & AT5G02260 | EXPA |
| AT2G28950 & AT2G37640 | EXPA |
| AT2G37640 & AT3G55500 | EXPA |
| AT2G37640 & AT2G39700 | EXPA |
| AT2G37640 & AT5G02260 | EXPA |
| AT2G39700 & AT5G02260 | EXPA |
| AT2G39700 & AT3G55500 | EXPA |
| AT2G40610 & AT5G05290 | EXPA |
| AT2G45110 & AT3G60570 | EXPB |
| AT2G20750 & AT4G28250 | EXPB |

**Tandem duplicated genes of the expansin gene superfamily in *Arabidopsis*.**

| Subfamily | Chromosome | Tandem duplicated genes |
| --- | --- | --- |
| EXPB | 1 | AT1G65680, AT1G65681 |
| EXLA | 3 | AT3G45960, AT3G45970 |
| EXPA | 5 | AT5G39260, AT5G29270, AT5G29280, |
|  |  | AT5G29290, AT5G29300, AT5G29310 |
