# Supplementary material for: Soybean (Glycine max) expansin gene superfamily origins: segmental and tandem duplication events followed by divergent selection among subfamilies
Source: BMC Plant Biol. 2014 Apr 11;14:93. doi: 10.1186/1471-2229-14-93 (PMC4021193; doi:10.1186/1471-2229-14-93)
Supplement: Additional file 14 — Expansion pattern of the expansin gene superfamily in rice. [file 1471-2229-14-93-S14.docx]

Additional File 14: Expansion pattern of the expansin gene superfamily in rice.

**Segemental duplicated gene pairs:**

| Segemengal duplicaged gene pairs | Subfamily |
| --- | --- |
| Os01g0248900 & Os05g0276500 | EXPA |
| Os01g0823100 & Os03g0336400 | EXPA |
| Os03g0155700 & Os10g0439200 | EXPA |
| Os03g0336400 & Os05g0477600 | EXPA |
| Os02g0639500 & Os04g0530100 | EXPB |
| Os02g0658600 & Os04g0552000 | EXPB |
| Os03g0102500 & Os04g0552000 | EXPB |
| Os03g0106500 & Os10g0548600 | EXPB |
| Os03g0132200 & Os10g0542400 | EXLA |

**Tandem duplicated genes of the expansin gene superfamily in rice.**

| Subfamily | Chromosome | Tandem duplicaged genes |
| --- | --- | --- |
| EXPA | 1 | Os01g0248900, Os01g0249100 |
| EXPA | 2 | Os02g0267700, Os02g0267900 |
| EXPA | 2 | Os02g0268400, Os02g0268600 |
| EXPA | 3 | Os03g0155300, Os03g0155500, Os03g0155600 |
|  |  | Os03g0155900, Os03g0156000, Os03g0156300 |
| EXPA | 5 | Os05g0276500, Os05g0277000 |
| EXPA | 10 | Os10g0439100, Os10g0439200 |
| EXPB | 2 | Os02g0658600, Os02g0658800 |
| EXPB | 3 | Os03g0102500, Os03g0106700 |
| EXPB | 3 | Os03g0106800, Os03g0106900 |
| EXPB | 4 | Os04g0552000, Os04g0552200 |
| EXPB | 10 | Os10g0555600, Os10g0555700, Os10g0555900, Os10g0556100 |
